# Examination of conformational dynamics of AdiC transporter with fluorescence-polarization microscopy

**DOI:** 10.1085/jgp.202413709

**Published:** 2025-02-20

**Authors:** John H. Lewis, Yufeng Zhou, Zhe Lu

**Affiliations:** 1 Department of Physiology, Perelman School of Medicine, University of Pennsylvania, Philadelphia, PA, USA

## Abstract

To understand the mechanism underlying the ability of individual AdiC molecules to transport arginine and agmatine, we used a recently developed high-resolution single-molecule fluorescence-polarization microscopy method to investigate conformation-specific changes in the emission polarization of a bifunctional fluorophore attached to an AdiC molecule. With this capability, we resolved AdiC’s four conformations characterized by distinct spatial orientations in the absence or presence of the two substrates, and furthermore, each conformation’s two energetic states, totaling 24 states. From the lifetimes of individual states and state-to-state transition probabilities, we determined 60 rate constants characterizing the transitions and 4 K_D_ values characterizing the interactions of AdiC’s two sides with arginine and agmatine, quantitatively defining a 24-state model. This model satisfactorily predicts the observed Michaelis–Menten behaviors of AdiC. With the acquired temporal information and existing structural information, we illustrated how to build an experiment-based integrative 4D model to capture and exhibit the complex spatiotemporal mechanisms underlying facilitated transport of substrates. However, inconsistent with what is expected from the prevailing hypothesis that AdiC is a 1:1 exchanger, all observed conformations transitioned among themselves with or without the presence of substrates. To corroborate this unexpected finding, we performed radioactive flux assays and found that the results are also incompatible with the hypothesis. As a technical advance, we showed that a monofunctional and the standard bifunctional fluorophore labels report comparable spatial orientation information defined in a local frame of reference. Here, the successful determination of the complex conformation-kinetic mechanism of AdiC demonstrates the unprecedented resolving power of the present microscopy method.

## Teaser

Lewis et al. use fluorescence-polarization microscopy method to define a 24-state model of the conformational dynamics of the AdiC transporter that underlie its ability to transport the amino acid arginine and the metabolite agmatine through biological membranes.

## Introduction

Transporter molecules adopt four basic types of conformation, namely, externally open (E_o_), externally occluded (E_x_), internally open (I_o_), and internally occluded (I_x_) states (Post et al., 1972; Shi, 2013; Krammer and Prévost, 2019). Structural biological studies have revealed structural features underlying these different functional conformations of numerous transporters (Shi, 2013; Krammer and Prévost, 2019). Despite the tremendous progress in functional and structural studies of transporters, some important issues remain. Among them are how to more effectively dissect complex conformational kinetics of transporters, and how to relate the temporal information to the structures of individual states. Addressing these issues requires the capability to track the rapid conformational changes that occur in individual molecules often on an angstrom scale. Furthermore, the temporal measurement at each time point needs to contain the spatial information that specifically identifies the conformational state. Only with relatable kinetic and structural information, one can fully explain the behaviors of a protein molecule in four dimensions (4D).

Fortunately, as a protein molecule undergoes conformational changes, some of its secondary structures, e.g., α-helices spatially constrained by other secondary structures, inevitably adopt unique spatial orientations in each conformational state. Thus, conformational changes can be tracked by monitoring such an α-helix’s spatial orientation defined in a spherical coordinate system by the inclination and rotation angles (*θ* and *φ*).

To accomplish this task, one can use a polarization microscope to monitor the emission polarization change of a bifunctional rhodamine attached to a suitable α-helix (Sase et al., 1997; Ha et al., 1998; Warshaw et al., 1998; Adachi et al., 2000; Fourkas, 2001; Sosa et al., 2001; Forkey et al., 2003, 2005; Rosenberg et al., 2005; Beausang et al., 2008; Ohmachi et al., 2012; Lippert et al., 2017; Lewis and Lu, 2019a). The documented resolutions of this type of method had been ≥25° calculated as 2.5 times of the standard deviation (σ) of angle distribution (Forkey et al., 2005; Rosenberg et al., 2005; Ohmachi et al., 2012; Lippert et al., 2017). To put this resolution in perspective, the estimated median radius of proteins is ∼20 Å (Brocchieri and Karlin, 2005; Erickson, 2009), and a 1.7 or 3.5 Å change (in the chord distance) would correspond to a rotation of such a radius by only 5° or 10°. Our group increased the effective resolution of *θ* and *φ* to 5° and successfully detected the conformational changes in the individual molecules of a K^+^ channel’s soluble domain, which occur on angstrom-and-millisecond scales (Lewis and Lu, 2019a, 2019b, 2019c).

Subsequently, we extended this high-resolution polarization microscopy method to studying a membrane protein, namely, the AdiC transporter (Zhou et al., 2023). AdiC naturally facilitates the movement of environmental (L-)arginine (Arg^+^) into enterobacteria and the discharge of agmatine (Agm^2+^) enzymatically generated in a decarboxylation reaction of cytosolic Arg^+^ (Gong et al., 2003; Iyer et al., 2003; Foster, 2004; Fang et al., 2007; Krammer and Prévost, 2019). Elimination of the coproduct CO_2_ effectively extrudes proton. Thus, AdiC is a key component of a proton-extrusion system critical for pathogenic enterobacteria to pass a host’s gastric barrier, reaching its intestines. Here, we use AdiC to show how to use the microscopy method to investigate highly complex protein conformational kinetics.

## Materials and methods

### Chemical reagents

Lipids 1-palmitoyl-2-oleoyl-glycero-3-phosphocholine (POPC), 1-palmitoyl-2-oleoyl-sn-glycero-3-phosphoethanolamine (POPE), and 1-palmitoyl-2-oleoyl-sn-glycero-3-phospho-(1′-rac-glycerol) (POPG) were purchased from Avanti Polar Lipids; detergents n-dodecyl-β-D-maltopyranoside (DDM), lauryl maltose neopentyl glycol, sodium cholate, and 3-[(3-Cholamidopropyl)dimethylammonio]-1-propanesulfonate (CHAPS) from Anatrace; monohydrochloride L-[2,3,4-3H]-Arginine and Ultima Gold LLT liquid scintillation cocktail from Revvity; sephadex G-50 resin from Cytiva; bifunctional rhodamine bis-((N-iodoacetyl)-piperazinyl)-sulfonerhodamine from Invitrogen (B10621); and ATTO-550 from ATTO-TEC GmbH; coverslip (#1.5) and microscope slide glass from Thermo Fisher Scientific or VWR; the MSP2N2 cDNA from Addgene (Plasmid #29520) (Grinkova et al., 2010). The AdiC cDNA was synthesized by Integrated DNA Technologies (Zhou et al., 2023). Unless specified otherwise, all other reagents were purchased from Sigma-Aldrich, Thermo Fisher Scientific, or EMD Millipore.

### Protein sample preparation, intensity recordings, and data reduction

The following technical aspects have been documented in recent publications (Lewis and Lu, 2019a, 2019b, 2019c; Zhou et al., 2023), which include sample preparation and validation, description of the polarization microscope, the intensity recordings, detection of concurrent changes in the intensities recorded in all four polarization channels using a specific changepoint algorithm, angle calculations, and identification of conformational states using a k-means–clustering algorithm on the basis of spatial orientations of the attached bifunctional rhodamine probe. The analyses were carried out in LabView with the analytic solutions given in the above references. Following a summary of these methods, we will describe the radioactive flux assay. Additional necessary analysis and calculation methods used in this study, which were performed with the analytical solutions in Mathcad, are described in the supplemental text at the end of the PDF.

The recombinant protein AdiC, which had a biotin moiety linked covalently to the N-terminal biotin ligase recognition sequence and strep tags in both N- and C-terminal regions, the double G188C and S195C cysteine mutations, or a single G188C mutation in helix 6A, and the double C238A and C281A mutation to remove these interfering native cysteines, was produced using the bacterial BL-21 expression system. Purified AdiC, in a buffer containing 50 mM Tris (pH 8), 100 mM NaCl, 2 mM tris(2-carboxyethyl)phosphine, and 2 mM DDM, was mixed with a lipid stock containing 50 mM POPC, 100 mM sodium cholate, 100 mM NaCl, and 20 mM Tris (pH 8), with a molar ratio of 1 AdiC dimer to 1500 POPC molecules. The mixture was rotated at 4°C for at least 1 h. Then, the recombinant MSP2N2 protein in a buffer containing 50 mM Tris (pH 8), 0.5 mM EDTA, 1 mM dithiothreitol (DTT), and 13 mM sodium cholate were added to the mixture to achieve a 1:10 ratio between dimeric AdiC and MSP2N2 (Grinkova et al., 2010). The mixture was further rotated at 4°C for at least an additional hour before detergents were removed from the sample by overnight dialysis at 4°C against an ice-cold buffer containing 100 mM NaCl and 20 mM Tris (pH 8). After dialysis, the AdiC protein in nanodiscs was labeled with biotin and bifunctional rhodamine via the double mutant cysteine residues or monofunctional fluorophore ATTO-550 via the single one. The resulting sample was repurified with size exclusion chromatography as previously described (Zhou et al., 2023).

For fluorescence polarization measurement, the individual labeled AdiC protein molecules, each of which was inserted into a nanodisc, were attached to streptavidin molecules via biotinylated N-termini and strep-tags in both N- and C-terminal regions, streptavidin molecules that had adhered to the surface of a polylysine-coated coverslip. While the sample protein was immersed in a solution (pH 5 unless specified otherwise) containing 100 mM NaCl, 100 mM DTT and 50 mM acetic acid, without or with Arg^+^ or Agm^2+^ at a specific concentration, polarized emissions from individual fluorescent labels, excited in the evanescent field created at the surface of the sample coverslip by a circularly polarized laser beam (532 nm), were collected using a fluorescence microscope (Nikon Ti-E) with a 100× oil achromatic objective of a numerical aperture of 1.49, sorted into four polarization emission channels, recorded by an electron-multiplying charge-coupled device (EMCCD) camera (Andor Ixon Ultra 897), and then acquired using the Nikon NIS-Elements software into a Dell PC and stored on its hard drive (Zhou et al., 2023). We summed up the values of individual pixels of a given fluorophore image to obtain the aggregated intensity value, without further analyzing the detailed spatial features of the image itself (Lewis and Lu, 2019a).

I_tot_, θ, φ, and Ω were calculated using Eqs. 11, 12, 13, and 33 in our recent publication (Zhou et al., 2023), respectively. Conformational transitions and states were sequentially identified in two separate steps. A changepoint algorithm (Chen and Gupta, 2001; Beausang et al., 2008) was applied to the intensity traces to detect the transitions between conformational events (Lewis and Lu, 2019a), whereas a shortest-distance-based algorithm (Press et al., 2007) was used to identify the conformational states of individual events on the basis of the information regarding *θ* and *φ* angles (Lewis and Lu, 2019a). However, operationally, we performed the shortest distance analysis in a Cartesian coordinate system where the x, y, and z values were calculated from the *θ* and *φ* angles along with a unit radius *r* (Zhou et al., 2023). To correct for underestimation of *θ*_L_, which stemmed from the wobble motion of the fluorophore dipole, we determined the wobble angle δ through a separate ensemble anisotropy study, as previously described (Lewis and Lu, 2019a). Bifunctional rhodamine–labeled AdiC in nanodiscs was diluted to a final concentration of 40 nM in buffers containing PBS (pH 7.4) and 0–80% glycerol. Fluorescence anisotropy for each sample was measured using a Photon Technology Instruments QuantaMaster fluorometer (Horiba) with 545-nm excitation and 575-nm emission wavelengths. The same protocol was used for ATTO550-labeled AdiC suspended in the detergent lauryl maltose neopentyl glycol (0.1 mM). The resulting δ value was 27.03° for the bifunctional fluorophore and 39.37° for the monofunctional fluorophore.

The fluorescence polarization experiments were performed on 10 separate occasions. Data acquired among these separate collections are statistically comparable and were pooled together, resulting in sufficiently narrow distributions, comparable with those previously illustrated (Zhou et al., 2023). The width of the distributions reflects both technical and biological variations. Outlier data were excluded on the following basis. First, particles whose total intensity exhibited more than one step-bleaching step were excluded. Second, for a given recording, at least 15 events are required to obtain a 95% confidence level for state identification, so any traces with <15 events were excluded on the assumption that the short and long traces belong to the same distribution. Third, for event detection and state identification, a signal-to-noise ratio (SNR) >5 is necessary for the required minimum angle resolution.

### Protein reconstitution into lipid vesicles

The lipids POPE and POPG in chloroform were mixed in a 3:1 (wt:wt) ratio, dried using a Buchi Rotavapor R-210 evaporator, stored under vacuum overnight, and resuspended to a final concentration of 20 mg/ml in a pH 7.4 buffer containing 100 mM NaCl, 4 mM N-methyl-D-glucamine (NMG), 1 mM tris(2-carboxyethyl)phosphine, and 10 mM Hepes. The mixture was sonicated in a water bath and mixed with CHAPS to a final concentration of 34 mM. After this mixture was rotated with a tube rotator for >2 h at room temperature, purified AdiC was added to it to achieve an AdiC to lipid ratio of 1:100 (wt:wt). The resulting mixture was rotated for an additional 30 min and then dialyzed three times against a pH 7.4 buffer containing 100 mM NaCl, 4 mM NMG, 1 mM β-mercaptoethanol, and 10 mM HEPES at room temperature before storing at −80°C.

### Arg^+^ uptake assay

Liposomes embedded with AdiC were dialyzed against a buffer containing 20 mM citric acid, titrated to pH 5.0 with KOH, and supplemented with the desired concentration of Arg^+^-HCl and KCl to a combined final concentration of 150 mM. The sample underwent three cycles of freezing and thawing, followed by sonication until becoming homogeneous, and was then passed through Zeba Spin Desalting Columns (2 ml, 40K MWCO; Pierce) equilibrated in the assay buffer containing 100–150 mM NaCl or KCl, 20 mM citric acid titrated to pH 5.0 with NaOH or KOH. This desalting process was repeated as necessary. Immediately after desalting, Arg^+^-HCl was added to achieve the desired concentration while maintaining constant ionic strength, and ^3^H-Arg^+^ was then added to the sample to a final concentration of 1–3 µCi/ml to initiate the uptake reaction (see below for desalting efficiency). At each time point, 50 μl aliquots of the sample were loaded onto 2.5-ml Sephadex G-50 columns (packed in-house), equilibrated in the assay buffer, and eluted with 1.2 ml of the same buffer into scintillation vials (see below for recovery efficiency of AdiC-containing liposomes). The eluates were mixed with liquid scintillation cocktail and counted using a Beckman LS6500 scintillation counter.

To measure Arg^+^ uptake into liposomes containing no substrate, reconstitution of AdiC into liposomes was performed in 150 mM KCl and 10 mM citirc acid, titrated to pH 5.5. Liposomes with AdiC were passed through Zeba Spin Desalting columns equilibrated in 150 mM NaCl and 10 mM citric acid, pH 5.5. Immediately after desalting, a mixture of unlabeled and ^3^H-labeled Arg^+^ was added to the sample to a final concentration of 2 mM for Arg^+^ and 1 µCi/ml for ^3^H-Arg^+^. Following incubation at room temperature for 2.5 h, 50 μl aliquots of the sample were processed as described above. Liposomes without AdiC were processed in parallel as negative, background controls.

### Efficiency of desalting columns

^3^H-Arg^+^ was added to the assay buffer to a final concentration of 4 µCi/ml. Two 650 μl aliquots were passed through two Zeba Spin Desalting Columns of 2 ml, equilibrated in the assay buffer. For each sample, a 50 μl aliquot was taken before and after passage through the columns, mixed with scintillation cocktail, and counted. The ratio of the radioactive counts in the samples before and after desalting gives the desalting efficiency. The desalting columns had an average efficiency of 99.9%. This high efficiency allowed us to accurately set the concentration of Arg^+^ in the bathing solution.

### Liposome recovery efficiency of G-50 columns

The experiment was conducted using separate G-50 columns. For each of the three trials, an Arg^+^ uptake assay was carried out as described above. 90 min after the uptake began, each sample of 50 μl was loaded onto a G-50 column and eluted with 1.2 ml of the assay buffer. In parallel, another 50 μl sample, as a control, was diluted with 1.2 ml of the assay buffer without going through the G-50 column. Subsequently, 125 μl of each sample that was eluted from the column or was simply diluted was passed through a Zeba Spin Desalting Column of 0.5 ml (40K MWCO; Pierce) equilibrated in the assay buffer. Individual samples flowing through from the Zeba columns were mixed with scintillation cocktail and then counted using a scintillation counter. The ratio of the two compared samples yielded an average recovery efficiency of 78% (±1%). This efficiency was used to calculate the actual fraction of radioactive substrate that moved into the vesicles via AdiC.

### Volume ratio between the bathing solution and total liposomes

An aliquot of the liposomes prepared for an uptake assay was reserved for determining the volume ratio. The Arg^+^ concentration outside the liposomes was adjusted to match the concentration inside. ^3^H-Arg^+^ was added to a final concentration of 3 µCi/ml. After incubating for 4 h at room temperature, each sample of 50 μl was loaded onto a G-50 column, eluted, and counted using the same procedure as in the uptake assays. Under such a symmetric concentration condition, the observed maximal fraction of radioactivity, $F_{\max}^{obs}$, moved through AdiC into the liposomes is proportional to the combined volume of the liposomes, whereas the remaining fraction, $1-F_{\max}^{obs}$, is proportional to that of the bathing solution. The ratio of 1−*F*_*max*_ and *F*_*max*_ gives the outside-to-inside volume ratio, *r*_v_ (Eq. 11). In principle, each batch of reconstituted liposomes would have a different volume ratio due to experimental errors. However, we have obtained relatively constant *r*_v_ under the same conditions. The mean *r*_v_ value was 129 ± 8 for the liposomes containing the wild-type AdiC protein and 110 ± 7 for the liposomes containing the mutant protein.

### Statistics

All experimental data were presented as mean ± SEM. F-tests were used to evaluate single- versus double-exponential fits with the MATLAB-enabled maximum-likelihood estimation tool (Woody et al., 2016). The confidence intervals of simulation data were obtained through the Monte Carlo simulations, and σ is in turn calculated from 68% confidence intervals.

### Online supplemental material

**Figure S1.**
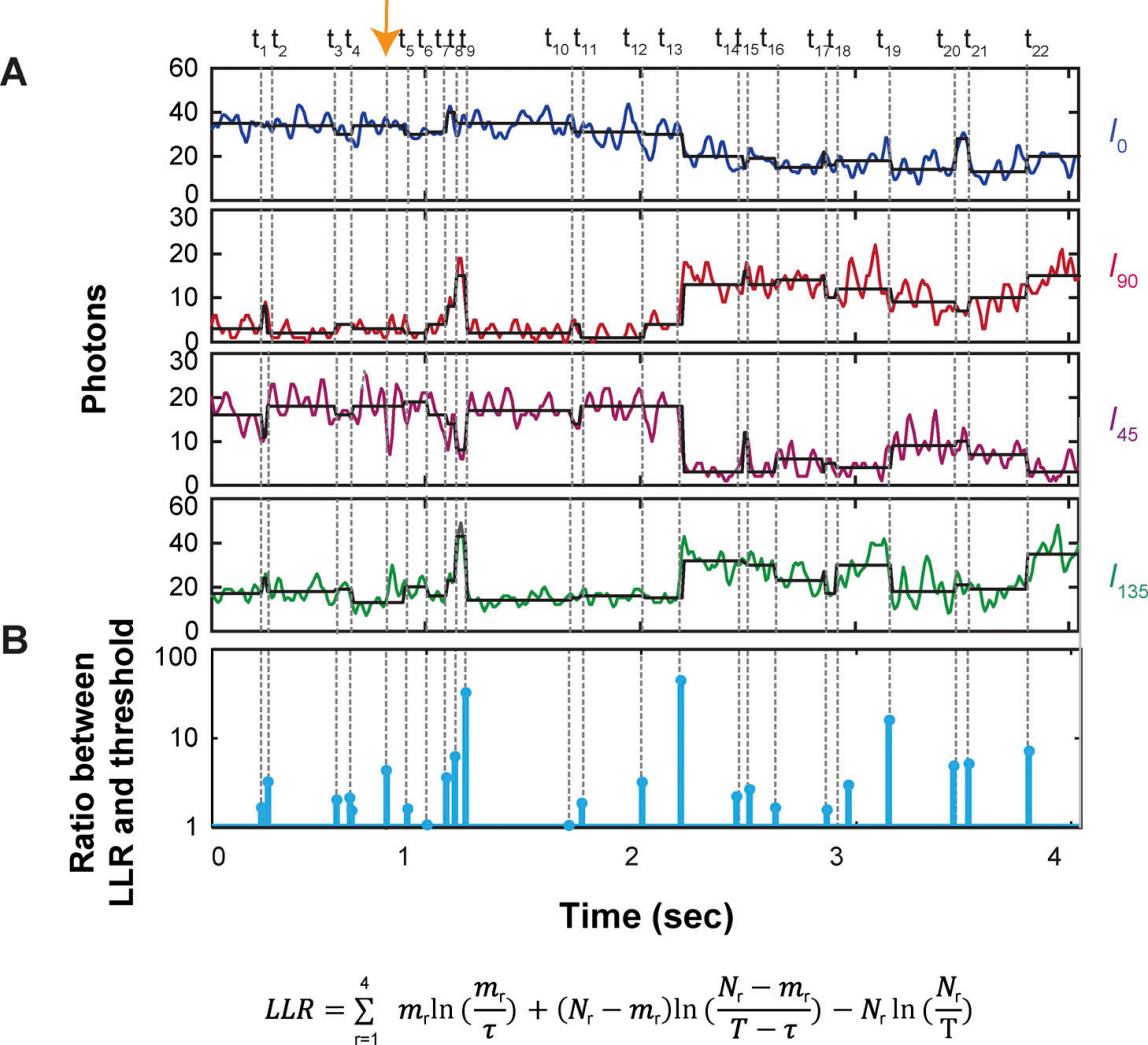
**Changepoint analysis of a set of polarized intensity components. (A)** The intensities of four polarized components are replotted from Fig. 1 A. Dashed vertical thin lines indicate individual finally identified intensity transitions, t_1_–t_22_, and a false positive one between t_4_–t_5_. **(B)** The ratio between LLR and the 95% confidence threshold is plotted for each identified transition. The equation to calculate LLR is given at the bottom (Lewis and Lu, 2019a), in which *m*_*r*_ is the number of photons detected during the duration *τ* from the beginning of a time segment and the evaluated time point, whereas *N*_*r*_ is the total number of photons detected during the total duration *T* of the segment. The final LLR is the sum of those for the individual four polarized components of intensity. As expected, all values are above one. By definition, for a 95% confidence detection threshold, 1 false positive occurs for every 20 identified transitions on average. Here, among the total 23 positives, the one pointed at by the orange arrow is judged to be false on the basis that it is a transition within *C*_1_ itself (see Results). Consequently, the number of finally identified intensity transitions is reduced by one to 22. LLR: log maximum likelihood ratio.

**Figure S2.**
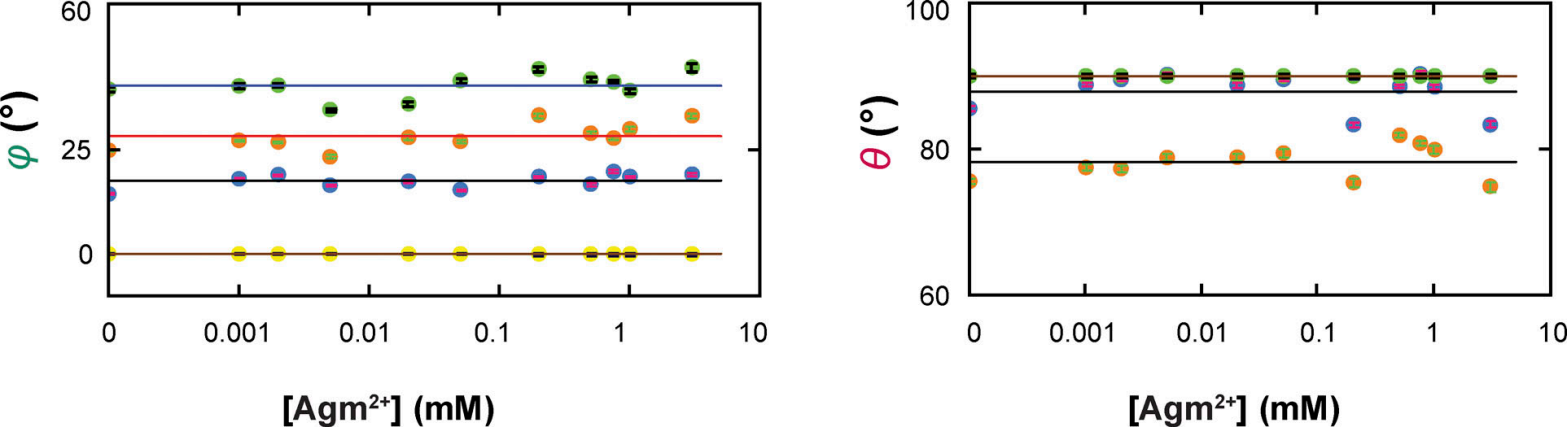
**Average angle values of individual conformational states in a series of Agm** ^**2*+***^ **concentrations.** The values of *θ* and *φ* (mean ± SEM) for each of the four conformations are plotted against the concentration of Agm^2+^. The number of events is 945–3,187. The symbols for the conformational state *C*_1_ are colored yellow, *C*_2_ colored blue, *C*_3_ colored orange, and *C*_4_ colored cyan. Note that as described in Results, mean *θ* for either *C*_1_ (yellow) or *C*_4_ (cyan) are set to 90° and are thus overlapped.

**Figure S3.**
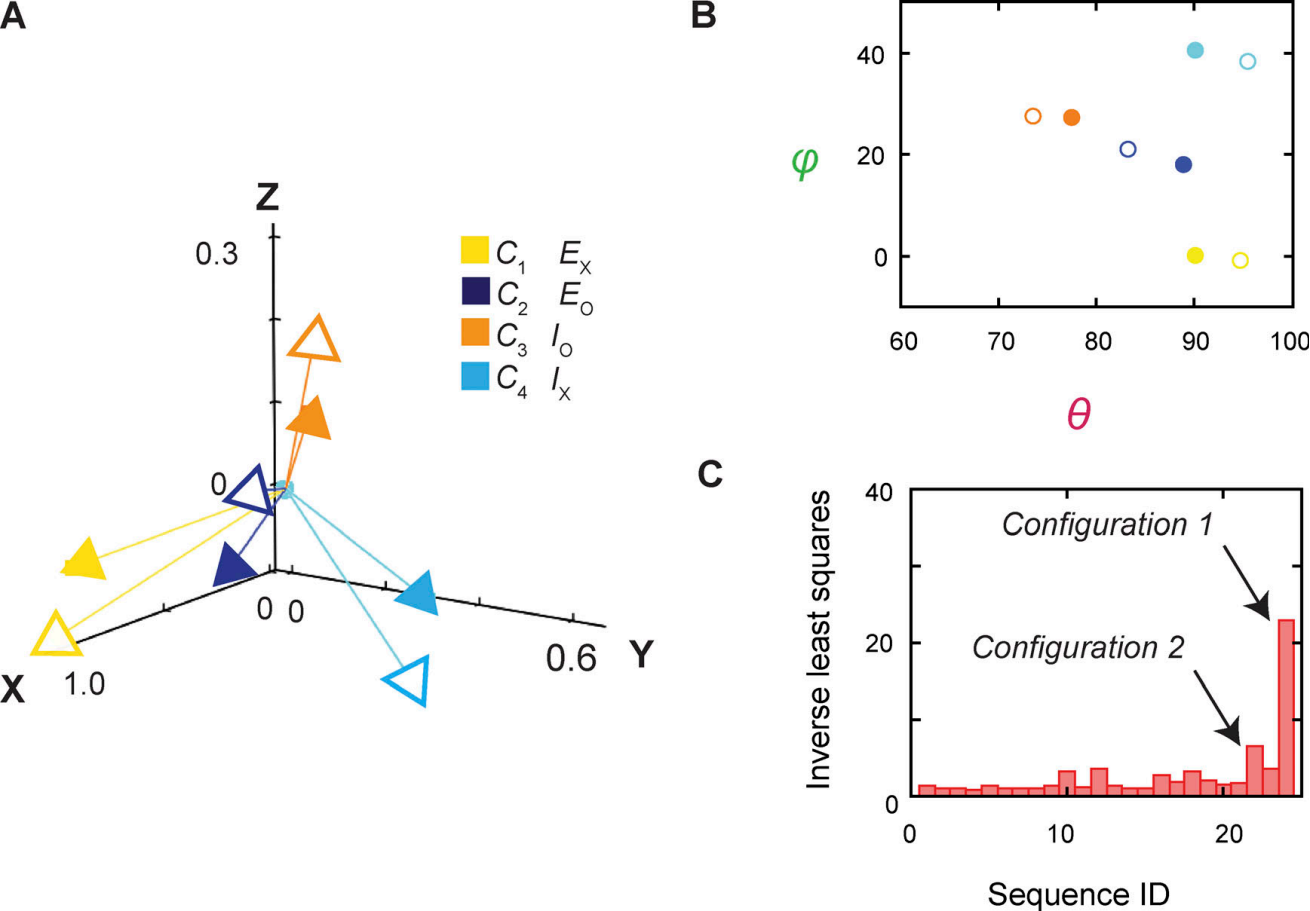
**Relations between the structural and conformational states determined, respectively, from the crystal structures and in the polarization study. (A)** The four mean orientations of the tracked helix in the four conformational states, determined over the examined range of the Agm^2+^ concentration, are represented by a set of four unit vectors (closed heads) in the local coordinates, whereas those for four structural states by another set of unit vectors (open heads), which are E_O_ (blue) (PDB: 7O82) and E_X_ (yellow) (PDB: 3L1L) of AdiC, I_O_ of BasC (orange) (PDB: 6F2G), and I_X_ of ApcT (cyan) (PDB: 3GIA). The vectors for the conformational states, color coded for the states, are drawn according to *θ* and *φ* values obtained from the corresponding distributions. The two sets of vectors are overlaid according to the combined least-distance squares. **(B)** Scatter plots of mean *θ* versus *φ* values for four conformational states (closed circles), which are compared with those for the four structural states (open circles); all are color coded for states. **(C)** The inverse values of combined least-distance squares between the locations of the arrow heads of the two compared groups (open versus closed) in panel A plotted against the identification (ID) numbers for all 24 possible combination sequences among them. The two cases corresponding to configurations 1 and 2 are indicated by the arrows.

**Figure S4.**
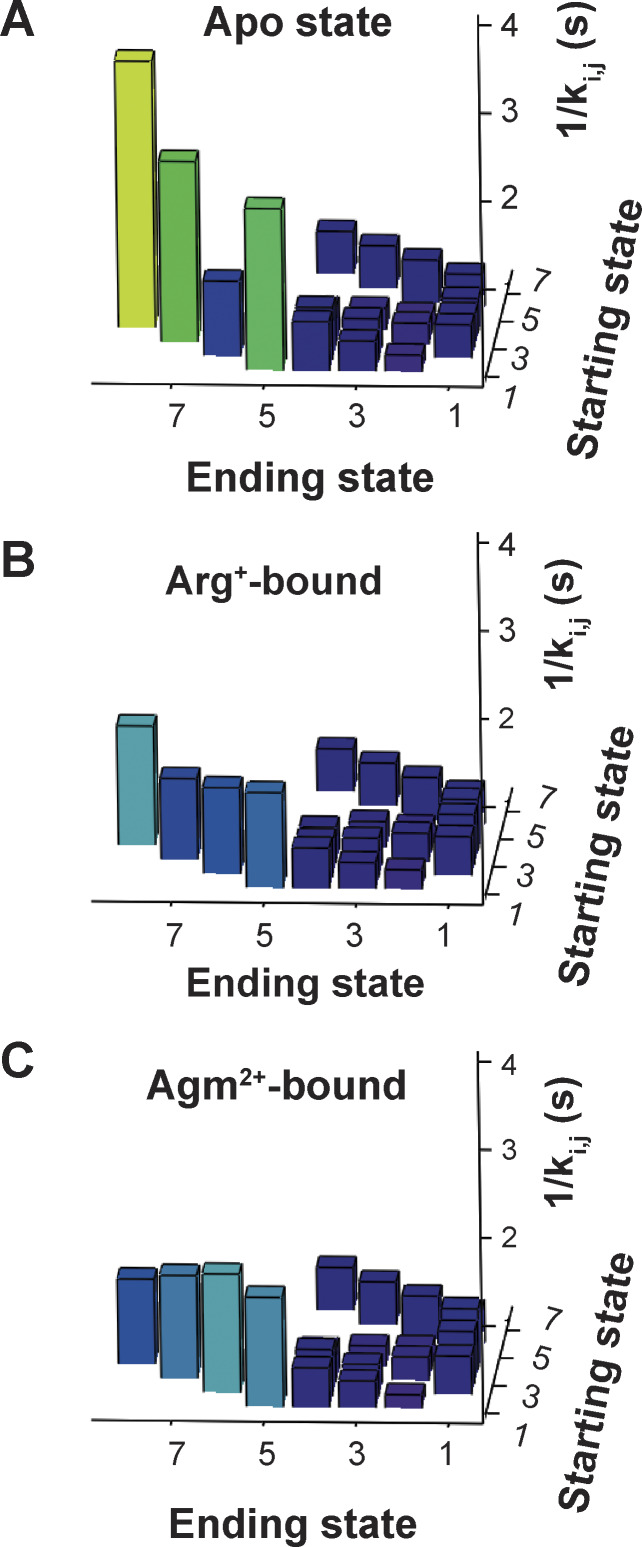
**Comparison of state-to-state transition rate constants of AdiC. (A–C)** The reciprocals of the rate constants (1/*k*_i,j_) plotted against the starting state *i* and the ending state *j*. The four taller columns correspond to the reciprocals of *k*_1,5_, *k*_2,6_, *k*_3,7_, and *k*_4,8_ in the absence (A) or the presence of Arg^+^ (B) or Agm^2+^ (C). The columns are color coded according to their height, namely, changing from blue toward yellow as the height increases.

**Figure S5.**
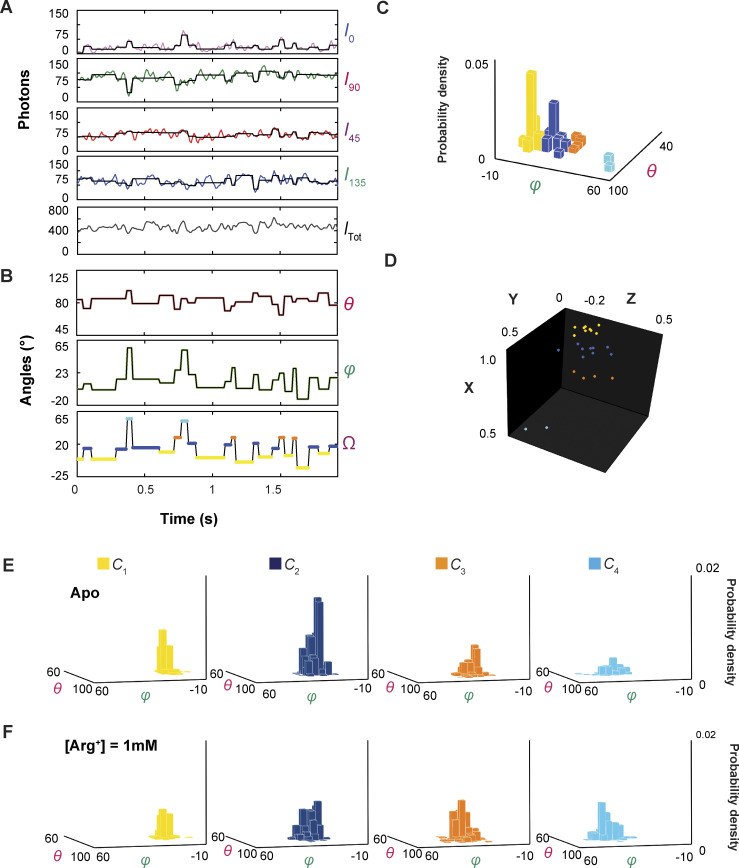
**The behaviors of a monofunctional fluorophore–attached helix 6A.** **(A)** The intensities recorded from a monofunctional fluorophore attached to an AdiC molecule in the absence of any substrate. Individual components are color coded and plotted against the observation time. Each black vertical line in the black traces, superimposed on the colored intensity traces, indicates the time point at which a change in the intensities and thus fluorophore’s orientation is identified, whereas each black horizontal line represents the mean intensity between two identified consecutive time points. **(B)** Traces for *θ* and *φ* are calculated from the black traces in A and expressed in the local frame of reference defined in Results. Values for the *Ω* trace are calculated from the *θ* and *φ* traces relative to the mean values for *C*_1_, where the states are color coded. **(C)** The 3D probability densities of individual conformational states plotted against *θ* and *φ* values. The *θ* and *φ* values for individual events are determined from the particle exhibited in panel A. **(D)** The positions of the arrowheads of individual vectors that represents the orientations of the fluorophore dipole in the individual events of adopting various conformations are mapped onto a unit sphere defined by the Cartesian coordinates of the local framework described in Results. The x, y, and z positions are calculated from *θ* and *φ* plotted in panel C, with a radius of a unit length that carry no physical meaning here. **(E and F)** Ensemble 3D probability density distributions of *θ* and *φ* in the absence (apo) and the presence of 1 mM Arg^*+*^. The distributions were built with the data analyzed from 25 to 36 particles with a total of 584–699 events. The histograms (C, E, and F) and data points (D) for conformational state *C*_1_ are colored yellow, *C*_2_ colored blue, *C*_3_ colored orange, and *C*_4_ color cyan.

**Figure S6.**
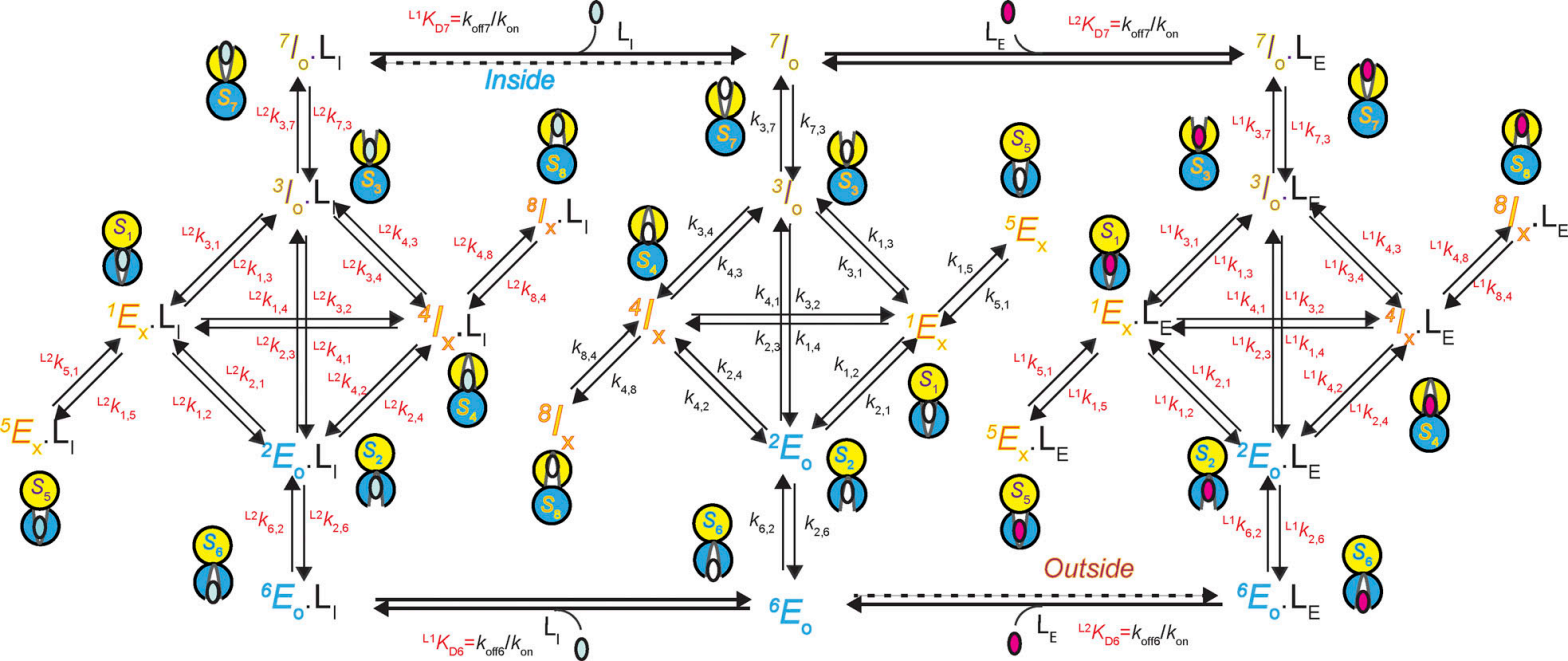
**A 24-state kinetic model of AdiC for transporting two types of ligand on opposite sides of the membrane.** All states are denoted according to the sidedness configuration 1 described in Results. As an example, S_6_ identified in the polarization study, which corresponds to the structural and functional state E_O_, is denoted as ^6^E_O_ (bottom middle). The middle portion described the transition of apo AdiC among eight identified states differing in conformation or energy. The left and right portions describe the transport of the intracellular ligand L_I_, e.g., Agm^2+^ or a nonradioactive ligand, and the extracellular ligand L_E_, e.g., Arg^+^ or a radioactive ligand, respectively. As initial conditions, L_I_ is only present in the internal solution, whereas L_E_ is only present in the external solution. After being transported across the membrane, they are present on both sides. Transition rate constants *k*_i,j_ and *k*_j,i_, associated with arrows, indicate the reversible transitions between corresponding states *i* and *j* (Tables S1, S2, S3, and S4). For distinction, the transitions involving the binding and unbinding of a ligand are labeled with $K_{D}=k_{\text{off}}/k_{\text{on}}$.

**Figure S7.**
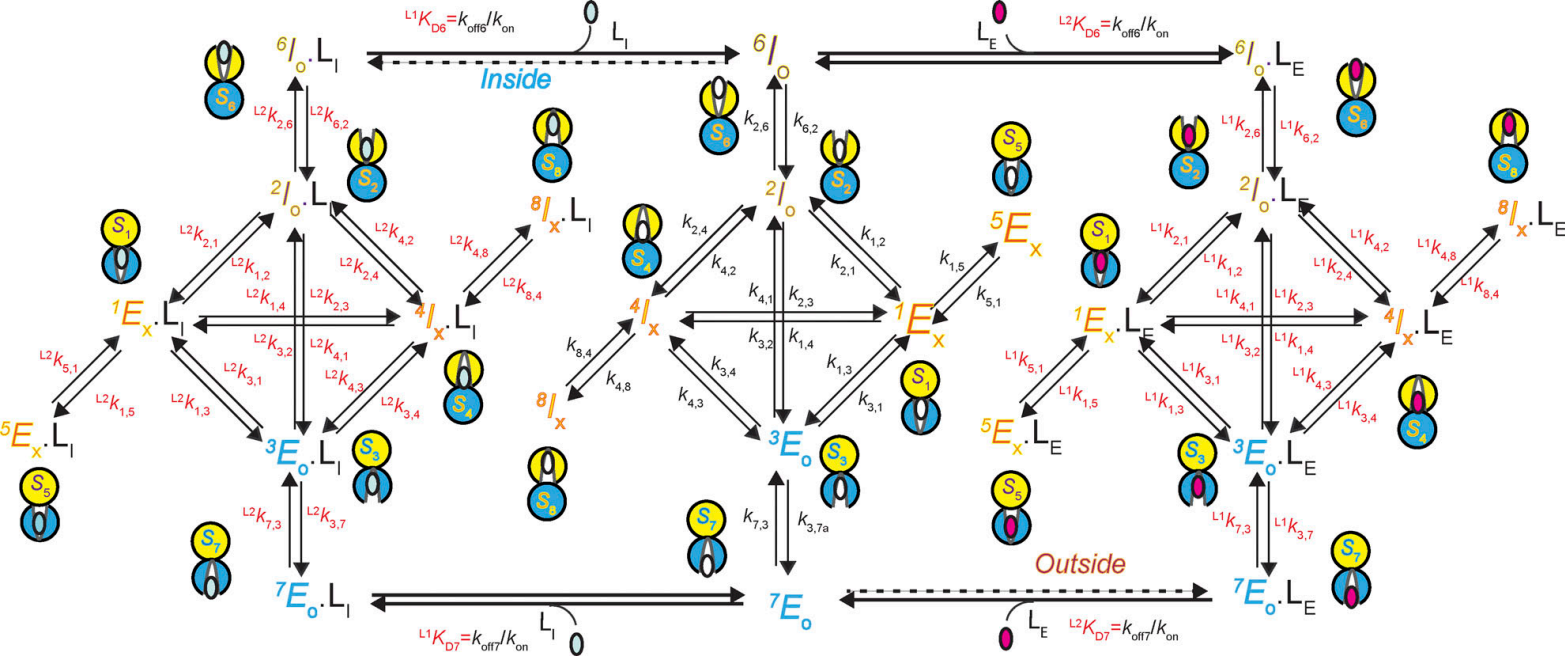
**An alternative 24-state kinetic model of AdiC for transporting two types of ligand on opposite sides of the membrane.** The model is the same as the one shown in Fig. S6, except that I_O_ and E_O_ are switched such that it becomes the sidedness configuration 2.

**Figure S8.**
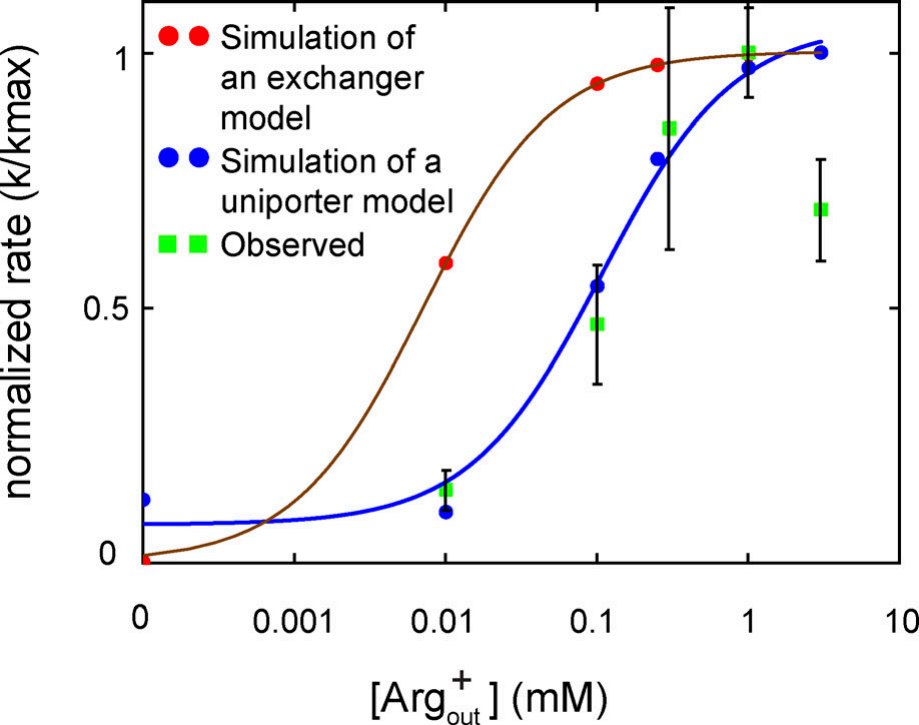
**Simulation of the dependence of the Agm** ^**2+**^ **-efflux rate from bacteria on the Arg** ^**+**^ **concentration in the medium.** The previously observed initial rate of Agm^2+^ extrusion at pH 2.5 (mean ± SEM; closed green squares) is normalized relative to the observed maximum and replotted here against the external Arg^+^ concentration on a logarithmic scale (Iyer et al., 2003). The simulations using the model (with kinetic parameters detemined in the polarization study at the corresponding pH) shown in Fig. S6 itself or with its apo path for conformational transitions eliminated, i.e., a uniporter or an exchanger model, are shown as closed blue or red circles (see supplemental text at the end of the PDF). The red curve is a fit of a Michaelis–Menten equation to the data simulated with the exchanger model, whereas the blue curve is a fit of a modified Michaelis–Menten equation to the data simulated with the uniporter model. The modification allows the rate to approach a nonzero minimum value as the external Arg^+^ concentration approaches zero.

**Figure S9.**
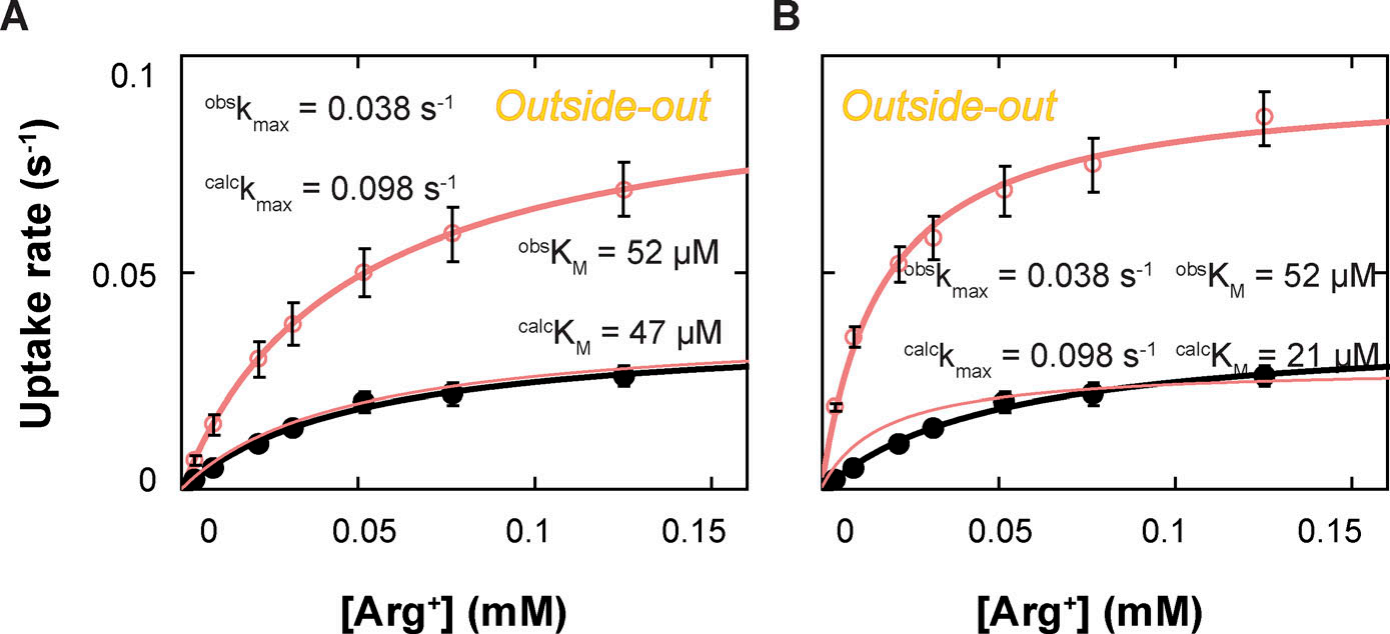
**Comparison of the observed and calculated rates of the AdiC-mediated uptake of Arg** ^**+**^ **into lipid vesicles in the outside-out configuration. (A and B)** Observed uptake rates of Arg^+^ (closed black circles; mean ± SEM) (Tsai et al., 2012) and the predicted rates by the model (open pink circles; mean ± SD), plotted against the Arg^+^ concentration. In panel A, *C*_2_ being assigned as E_o_ and *C*_3_ as I_o_ (Fig. S6), whereas in panel B, *C*_2_ as I_o_ and *C*_3_ as E_o_ (Fig. S7). The solid curves superimposed on symbols representing the calculated values correspond to the fits of the Michaelis–Menten equation. In the presence of near saturating concentrations of a given type of substrate on both sides of the membrane, the observed maximal net-flux rates (*k*_max_) are expected to be comparable in both orientations of AdiC. Indeed, under the three conditions that differ in substrate type or AdiC orientation, *k*_max_ values are comparable (Fig. 12). However, observed *k*_max_ for Arg^+^ in the outside-out orientation shown here is much lower, which reflects an underestimate of the maximal transporting rate or an overestimate of the effective number of AdiC molecules or both. Our calculations, as expected, yield comparable *k*_max_ values for all four conditions. The divergence between observed and calculated *k*_max_ values in the outside-out configuration is illustrated graphically by overlaying the observed data and the thin pink curve that represents a Michaelis–Menten equation fit to the calculated data in A or B after scaling down.

**Figure S10.**
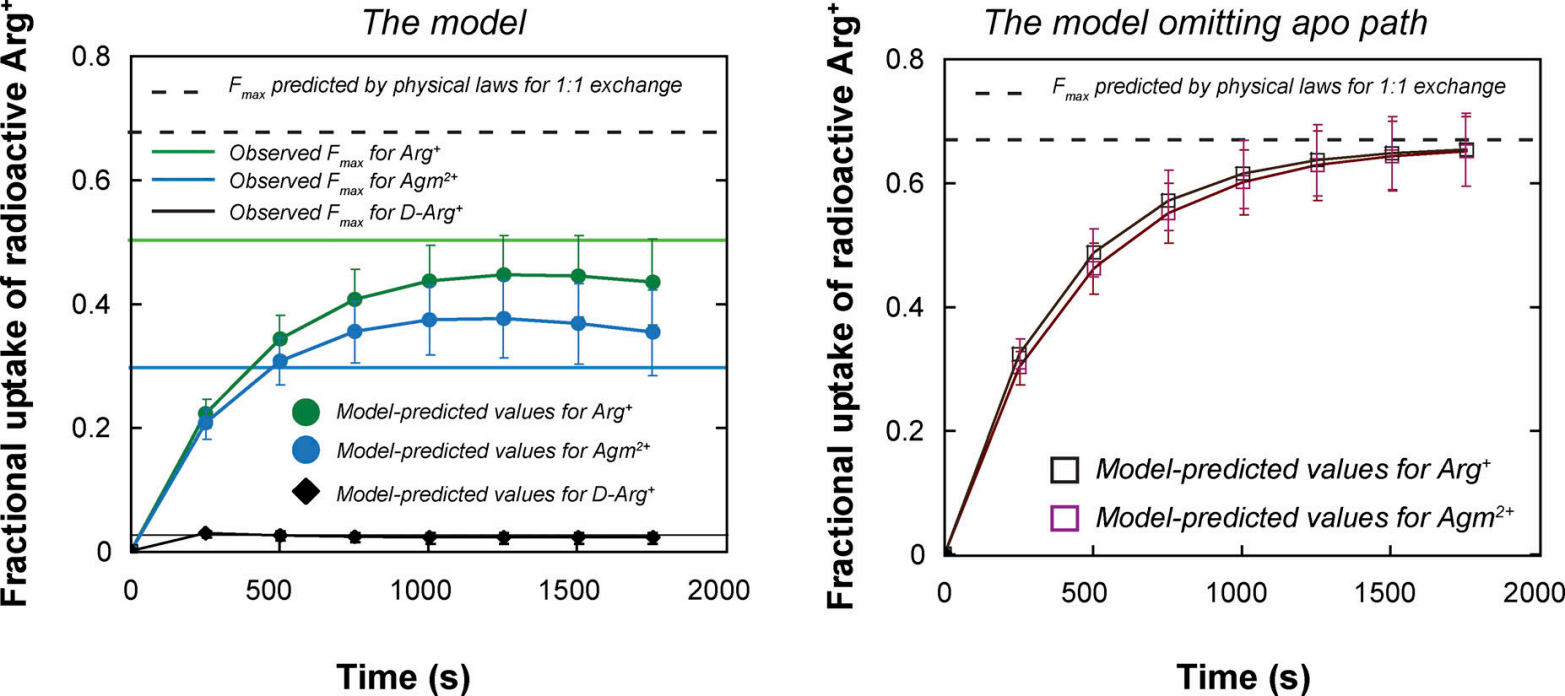
**Model-predicted uptakes of radioactive Arg** ^**+**^ **into AdiC-embedded lipid vesicles compared with the observed and theoretical maximum values. (A)** The model-predicted fractions of radioactive Arg^+^ (mean ± σ) accumulated inside the vesicles for individual indicated durations were obtained from the simulation with the model (Fig. S6), as shown in Fig. 11 A (see Results, and supplemental text at the end of the PDF), under the initial conditions that the bathing solution always contained 50 µM radioactive Arg^+^, whereas the vesicles contained 5 mM Agm^2+^ (closed blue circles), 5 mM Arg^+^ (closed green circles), or 5 mM functionally inert D-Arg^+^ (closed black circles), compared with the previously $F_{\max}^{obs}$ for the corresponding conditions indicated by the solid lines in the respective color (Fang et al., 2007). The dashed line at the top indicates the theoretical *F*_*max*_ for an obligatory 1:1 exchange under the initial conditions that the bathing solution always contained 50 µM radioactive Arg^+^, whereas the vesicles contained 5 mM Agm^2+^ or Arg^+^ (Eq. 10). **(B)** The model-predicted time-dependent fractions of radioactive Arg^+^ uptake (mean ± σ) were obtained from the simulation with the model after omitting the apo path for confirmational transitions, under the initial condition that the bathing solution always contained 50 µM radioactive Arg^+^, whereas the vesicles contained 5 mM Arg^+^ (open black squares) or Agm^2+^ (open maroon squares), compared with the theoretical *F*_*max*_ indicated by the black dashed line.

**Figure S11.**
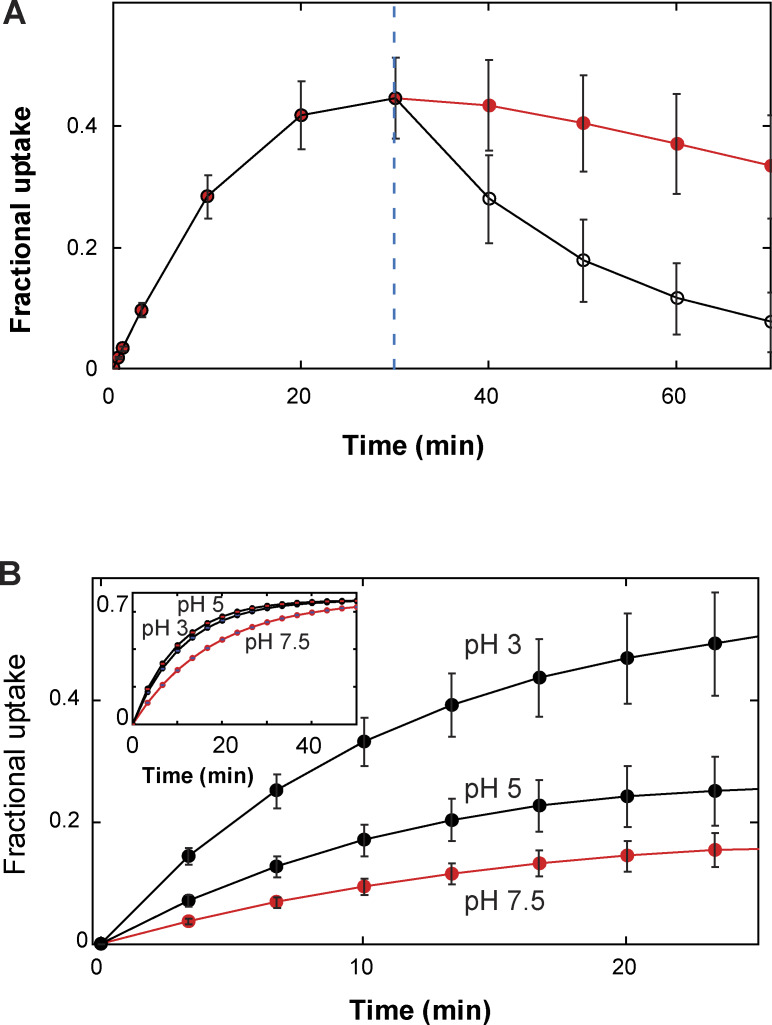
**Simulations of radioactive Arg** ^**+**^ **efflux induced by lowering the Arg** ^**+**^ **concentration in the bathing solution and the variation of fractional uptake of radioactive Arg** ^**+**^ **with a symmetric change in pH. (A and B)** The time courses of fractional uptake of radioactive Arg^+^ (mean ± σ) were simulated using our model with the parameters determined from the polarization measurements under the symmetric pH conditions. As the initial substrate conditions, the vesicles contained 5 mM Arg^+^, whereas the bathing solution contained 50 μM radioactive Arg^+^. The ratio of volumes of the bathing solution and vesicles was 50. **(A)** 30 min into the simulation, the calculation was continued either without any intervention (closed red circles) or with the Arg^+^ concentration of the bathing solution being raised to 5 mM (open black circles), the latter of which markedly lowered the fractional uptake in a time-dependent manner or induced an efflux of radioactive Arg^+^. **(B)** The simulations were performed with our model (of kinetic parameters detemined in the polarization study at the corresponding pH) under three conditions where pH was varied symmetrically on both sides of the membrane. For comparison, the simulations under the same conditions with the model whose apo path for conformational transitions was eliminated were shown in the inset. Note that Tsai et al. (2012) showed that mutant AdiC with native cysteines removed had slower kinetics than wild-type AdiC. As expected, the kinetics of the simulated time courses using our model with the kinetic parameters obtained from an AdiC mutant with native cysteines removed are slower than those of wild-type AdiC reported by Fang et al. (2007) but are comparable with those of the mutant AdiC reported by Tsai et al. (2012).

**Figure S12.**
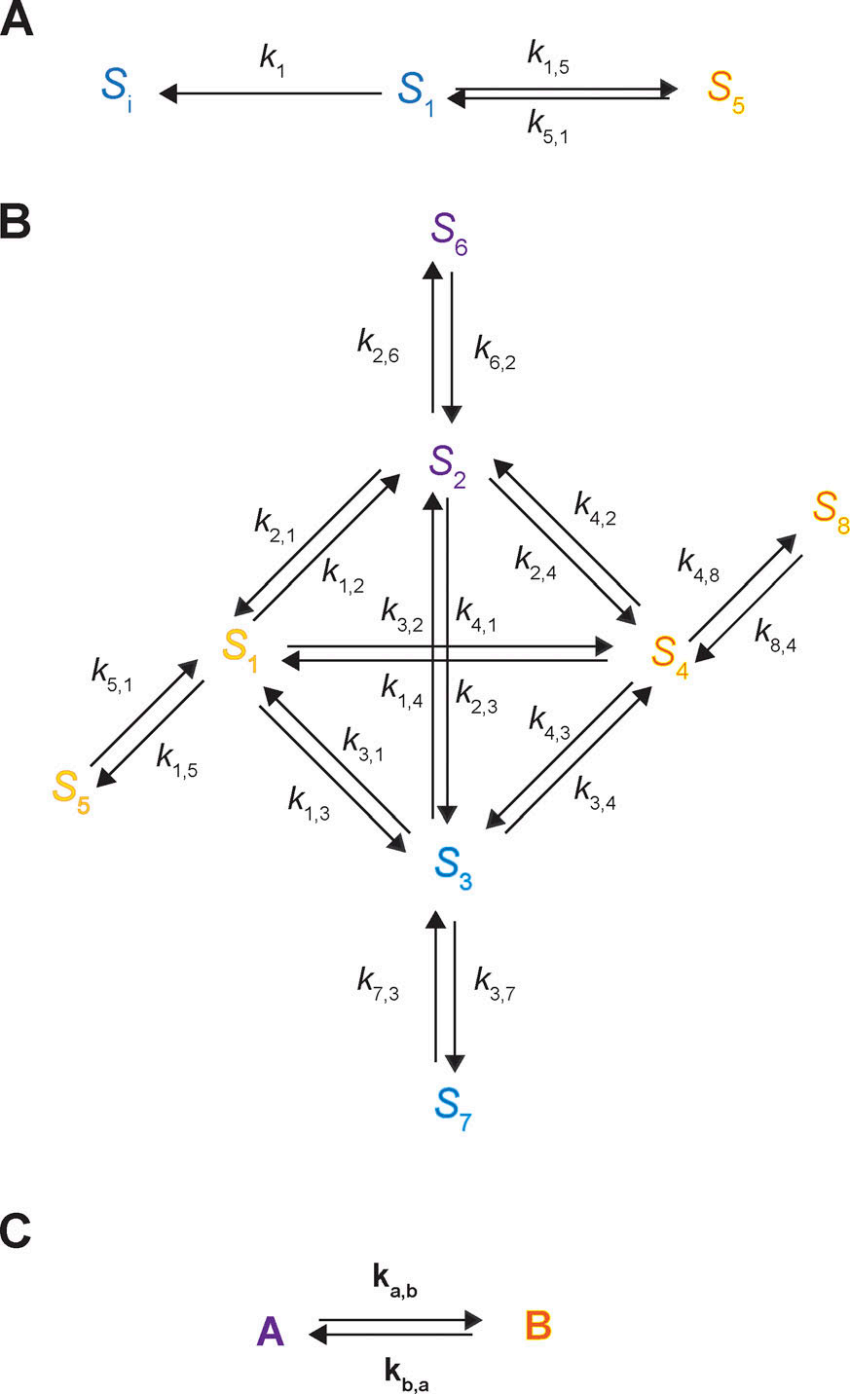
**Portions of the 24-state kinetic model. (A)** Diagram of the states *S*_1_ and *S*_5_ with the corresponding forward and backward rates *k*_1,5_ and *k*_5_, which are the energetic states underlying the conformation state *C*_1_. *S*_1_ also transitions to an ensemble of *S*_2_, *S*_3_, or *S*_4_, denoted as *S*_i_ with an apparent ensemble rate constant *k*_1_, as described in the supplemental text at the end of the PDF. **(B)** Diagram of the eight apo states *S*_1_ − *S*_8_, which corresponds to the middle section in Fig. 6. The transition matrix Q_apo_ corresponding to this diagram is given in Eq. S25. **(C)** The eight-state model in the panel B is reduced to a system with two ensemble-state components, A and B, where A comprises the states *S*_1_ − *S*_4,_ whereas B comprises the states *S*_5_ − *S*_8_. The corresponding transition matrix is given in Eq. S26.

Figs. S1, S2, S3, S4, S5, S6, S7, S8, S9, S10, S11, and S12 present additional necessary supporting data. Tables S1, S2, S3, S4, S5, S6, S7, S8, S9, S10, S11, S12, and S13 summarize the equilibrium and kinetic parameters yielded from analyzing the polarization data, or the Michaelis–Menton parameters of the previously observed or model-predicted transport-kinetic behaviors. Videos 1 and 2 exhibit the conformational changes of a single transporter subunit and the 4D model of its conformational dynamics. Supplemental text at the end of the PDF provides  the detailed kinetic analysis of data and the derivation of analytic expressions of the conformation-kinetic and transport-kinetic models.

## Results

### Recordings of fluorescence intensity and determination of fluorophore orientation

**Figure 1.**
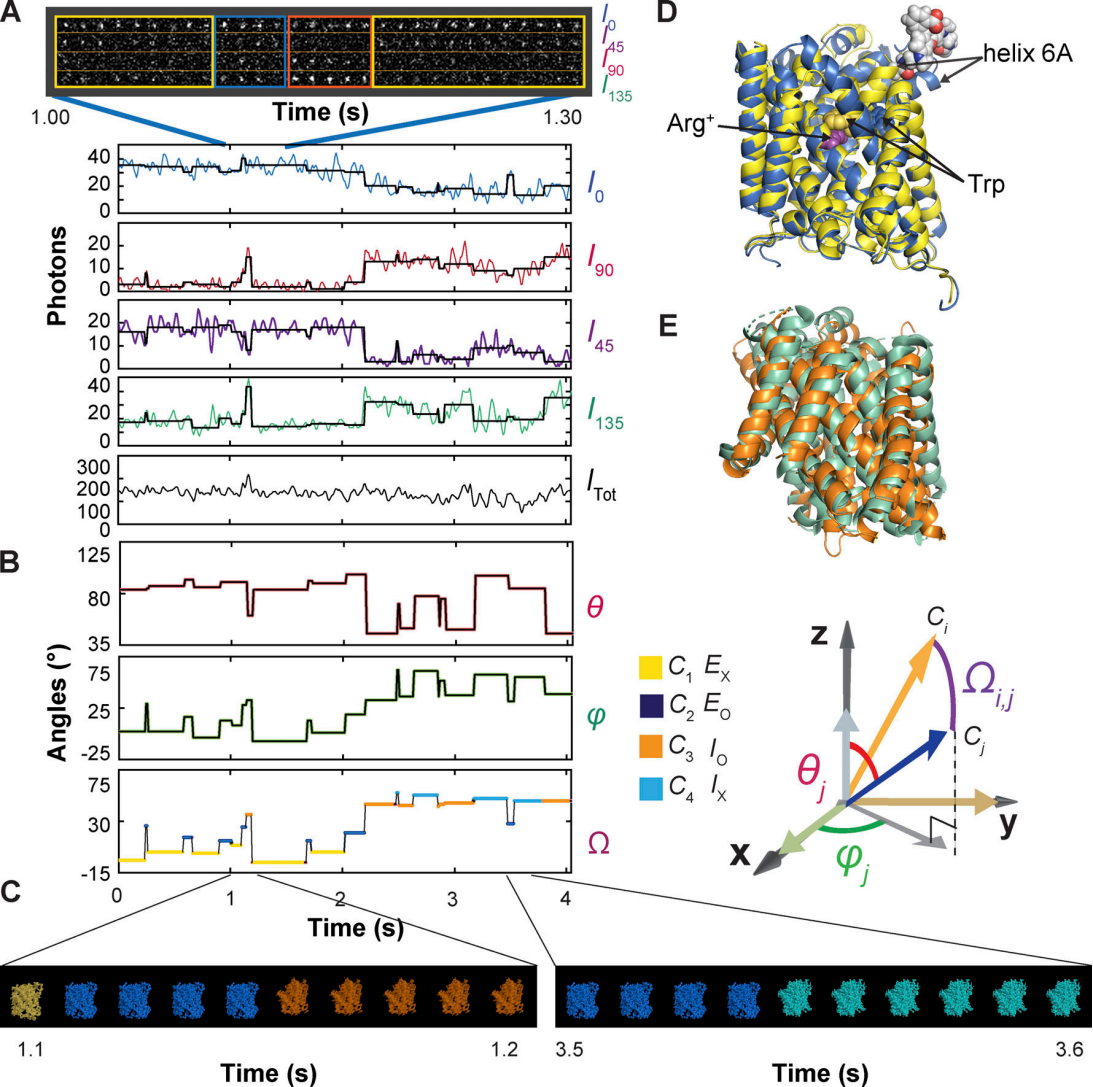
**Polarized intensity components of a single fluorescent particle, *θ* and *φ* angles calculated from these components, and segments of a video of conformational changes. (A)** A segment of consecutive frames of four intensity components (*I*_0_, *I*_45_, *I*_90_, and *I*_135_) of a bifunctional rhodamine label attached to an AdiC molecule, obtained in the presence of 50 μM Agm^2+^ and pH 5. The integrated values of the recorded intensities color coded for individual components and *I*_tot_ are plotted underneath against the observation time. Each vertical line in the black traces, superimposed on the colored intensity traces, indicates the time point at which a change in the fluorophore’s orientation is identified, whereas each horizontal line represents the mean intensity between two identified consecutive time points. **(B)** Traces for *θ* and *φ* are calculated from the black traces in A and expressed in the local frame of reference defined in Results. Values for the *Ω* trace are calculated from the *θ* and *φ* traces relative to the mean values for *C*_1_, where the states are color coded. The graphic definition of the angles is shown on the right. **(C)** Consecutive frames of two segments in Video 1 of AdiC conformational changes in which, as described in Results, the four conformations are represented by the corresponding electron density maps (PDB: 7O82, 3L1L, 3GIA, and 6F2G), in accordance with the temporal information encoded in the *Ω* trace, in which the state identities of individual events are color coded. **(D and E)** Exhibited in D are spatially aligned structures of E_X_ and E_O_ of AdiC shown with a single subunit (PDBs: 3OB6 and 3L1L), and in E those of I_O_ of BasC and I_X_ of ApcT (PDBs: 6F2G and 3GIA). Helix 6 in E_X_ of AdiC is attached with a bifunctional rhodamine represented with a space-filling model. The substrate Arg^*+*^ (purple) and a Trp residue (yellow) external to it in E_X_ of AdiC are represented using space-filling models. The Trp residue (blue) in E_O_ points to the opposite direction. Helix 6, Arg^+^, and the Trp residue are all identified by labeled arrows.

**Figure 2.**
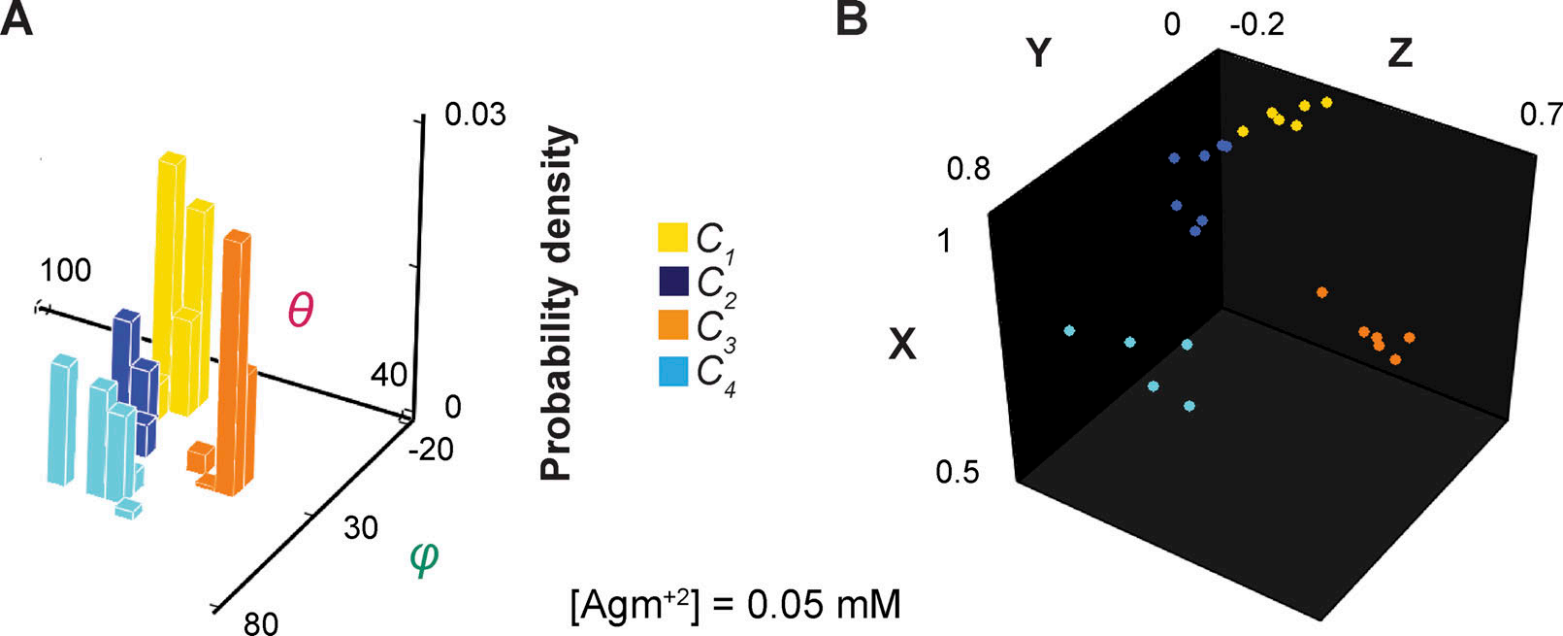
**The orientations of the dipole vector of the fluorescence probe in different conformational states in the local frame of reference. (A)** The 3D probability densities of individual conformational states plotted against *θ* and *φ* values. These angle values for individual events of adopting various conformations are determined from the particle exhibited in Fig. 1. **(B)** The positions of the arrowheads of individual vectors that represent the orientations of the fluorophore dipole in the individual events are mapped onto a unit sphere defined by the Cartesian coordinates of the local framework described in Results. The x, y, and z positions are calculated from *θ* and *φ* plotted in panel A, with a radius of a unit length that has no physical meaning here. The histograms (A) and data points (B) for conformational state *C*_1_ are colored yellow, *C*_2_ colored blue, *C*_3_ colored orange, and *C*_4_ color cyan.

**Figure 3.**
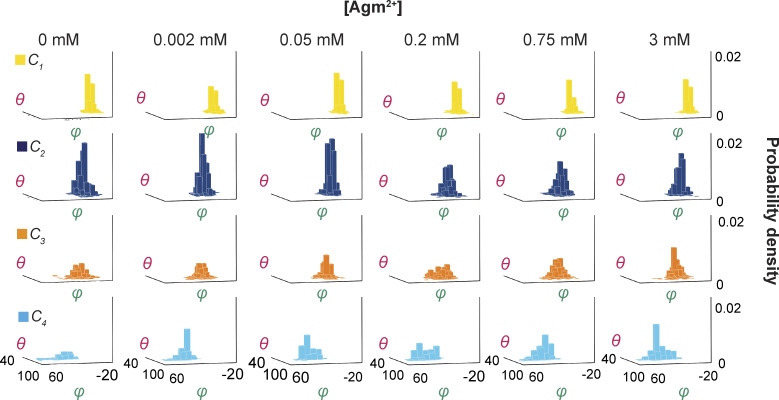
**Ensemble 3D probability density distributions of *θ* and *φ* in a series of Agm** ^**2*+***^ **concentrations.** The *θ* and *φ* distributions of four individual states in the absence or presence of the indicated concentrations of Agm^2*+*^, in which the value of *φ* is plotted along the x axis, *θ* along the y axis, and the probability density along the z axis. Distributions were built with the data analyzed from 34 to 91 particles with a total number (*n*) of 945–3,187 events. Histogram bars for the conformational state *C*_1_ are colored yellow, *C*_2_ colored blue, *C*_3_ colored orange, and *C*_4_ colored cyan.

**Figure 4.**
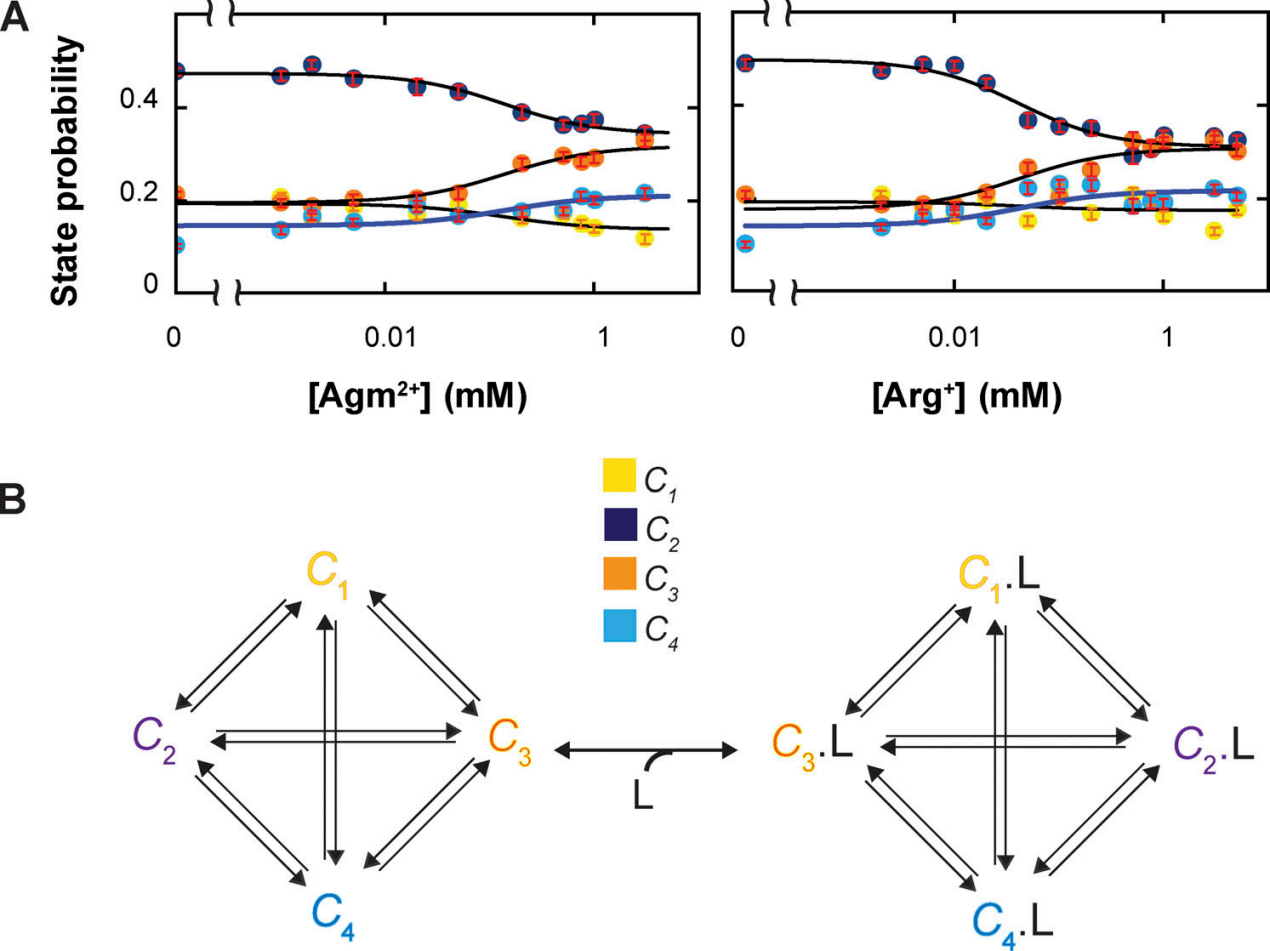
**Ligand dependence of the probabilities of conformational states and the diagram of a conformational state model of AdiC. (A)** The probabilities of individual four states (mean ± SEM, *n* = 945–3,187) are plotted against the Agm^2*+*^ concentration on a logarithm scale, along with those previously obtained with Arg^+^ on the right (Zhou et al., 2023). The curve superimposed on the data for a given conformation represents the predicted relation by the kinetic model (Fig. 6). **(B)** An eight-state model that accounts for the observed conformational behaviors of AdiC: four apo states and four Arg^+^- or Agm^2+^-bound states.

**Figure 5.**
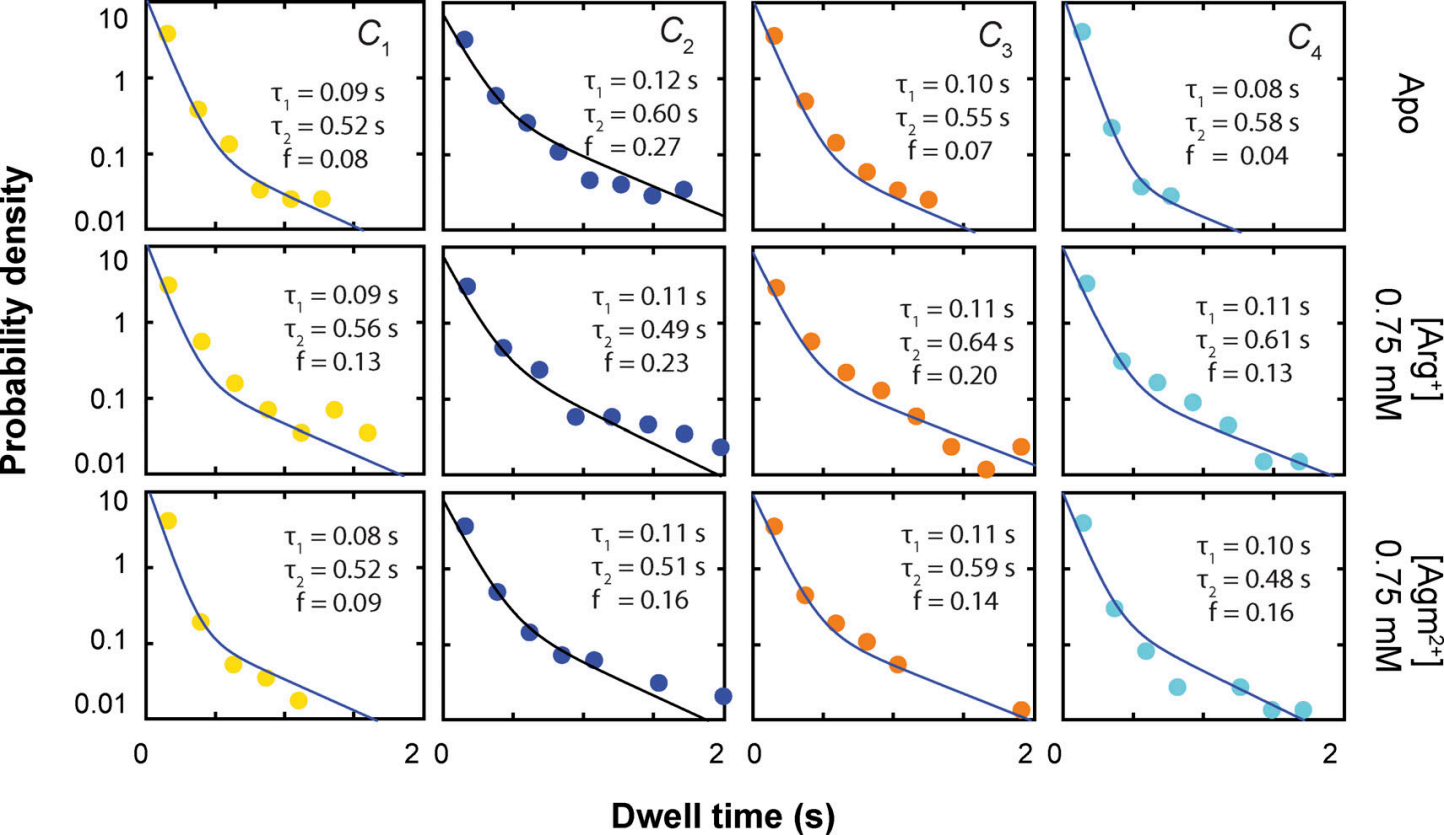
**Examples of dwell-time distributions of conformational states.** The distributions for *C*_1_–*C*_4_ in the absence or presence of 0.75 mM Arg^+^ or 0.75 mM Agm^2+^, plotted as the probability density on a log scale against the dwell time of a given state. Each distribution was built from 945 to 3,187 dwelling events. For each state, the fit was performed using a two-exponential equation (Eq. S1) to the data collected over all tested concentrations as a global operation, in which each component is coupled to a ligand-binding model expressed in the same form as Eq. 3.

We have previously shown the collection and reduction of data from individual AdiC molecules without or with its substrate Arg^+^ using the polarization microscopy method (Zhou et al., 2023). Because the present kinetic studies require also knowing the behaviors of AdiC bound with Agm^2+^, we will briefly go over the procedures for acquiring data from individual fluorescence-labeled AdiC molecules in the presence of Agm^2+^ and their analysis (Figs. 1, 2, 3, 4, and 5), before illustrating how to extract the kinetic information from the dwell times and state-to-state transition probabilities obtained with or without Arg^+^ or Agm^2+^.

We tracked the orientation changes of a portion of the AdiC molecule through monitoring the emission-polarization changes of a bifunctional rhodamine molecule attached to its helix 6A (Fig. 1 D). This helix adopts differing orientations in different known structural states, such as E_o_ and E_x_ (Fang et al., 2009; Gao et al., 2009, 2010; Kowalczyk et al., 2011; Ilgü et al., 2016, 2021). Helix 6A is attached by the fluorophore via two mutant cysteines, attachments aligning the fluorophore along the helix (Corrie et al., 1998). We chose this labeling site also for it being on the surface of AdiC, minimizing the label’s impact on AdiC function so that the acquired information can account for the function. Individual AdiC molecules were inserted into nanodiscs (Ritchie et al., 2009; Grinkova et al., 2010; Denisov et al., 2019), and attached to a coverslip with some flexibility to avoid over restrictions. Thus, the two-fold symmetry axis of individual dimeric AdiC molecules is not expected to align with or to have the same orientation relative to the *z*_L_ axis of the usual laboratory (L) frame of reference. Furthermore, the orientation of an AdiC molecule attached to the coverslip was random in the *x*_L_-*y*_L_ plane. To solve this problem, we computationally rotated all molecules into a common local frame of reference defined below. All references to AdiC’s conformational changes and functions are in the context of a single protomer.

The total intensity (*I*_tot_), recorded from an attached rhodamine in the presence of 50 µM Agm^2+^ during individual 10-ms sampling intervals, was sorted into four polarized components (*I*_0_, *I*_45_, *I*_90_, and *I*_135_; Fig. 1 A). Unless specified otherwise, all intensities measurements were obtained at pH 5 for comparison with existing flux-assay data (Tsai et al., 2012). Among their tested pH conditions, a greater fractional uptake occurred with symmetric pH of ∼5.

For reference, the relations of *φ* and *θ* with the four intensity components, derived for ideal conditions, are given below:

$$\varphi =\frac{1}{2}\tan^{-1}\frac{I_{45}-I_{135}}{I_{0}-{\;I}_{90}}$$ (1)

$$\theta =\sin^{-1}\left[2\sqrt{\frac{\sqrt{{\left(I_{0}-{\;I}_{90}\right)}^{2}+{\left(I_{45}-I_{135}\right)}^{2}}}{\left(I_{0}+{\;I}_{90}+I_{45}+I_{135}\right)}}\right]$$ (2)

When AdiC transitions between two conformations, the orientation of the attached fluorophore characteristically changes relative to the constant polarization angles of the two polarized beam splitters. For example, should the fluorophore move to increase its *φ* angle from 0 to 90°, *I*_0_ would decrease, whereas *I*_90_ would increase, while their sum should remain constant; *I*_45_ and *I*_135_ would also change in opposite directions. The concurrent changes in four components afford the opportunity for detecting intensity transitions with high confidence.

The vertical black lines superimposed on the recorded intensities indicate the individual time points at which intensity changes occurred (Fig. 1 A). These changes brought about by alterations in the fluorophore’s orientation were detected by an algorithm termed “changepoint” (Chen and Gupta, 2001), adopted here for our case (Lewis and Lu, 2019a). The algorithm tests the two possibilities that a change did or did not occur in the form of their log maximum likelihood ratio with 95% confidence. The program is started by identifying a single transition over the entire trace. If a transition were identified at time point X, it would then search for another single transition between the start of the trace and X or between X and the end. This iterative search process with successively shortened stretches continued until no more transitions were identified. To verify against false positives and refine the positions of individual transition points, we reexamined each identified transition point (e.g., transition t_i_) in the region demarcated by t_i-1_ and t_i+1_ (also see below). To check against false negatives, we examined each region demarcated by t_i_ and t_i+1_ to ensure that no new transitions were identified. For the example shown in Fig. 1 A, the ratio of log maximum likelihood ratio of each identified transition over the 95% confidence threshold expected for the noise level is presented in Fig. S1.

After acquiring this temporal information, averaging the intensities over each dwell time in a particular state markedly increased SNR and thus angle resolution. Each horizontal black line between a pair of consecutive vertical black lines superimposed over the intensity traces corresponds to the mean intensity value during the dwell time (Fig. 1 A). From the traces defined by those black lines, we calculated the angles *θ* and *φ*, and then the direct angle change *Ω* between two states (Fig. 1 B). To calculate *θ* and *φ*, we used the expanded versions of Eqs. 1 and 2 (Eqs. 11 and 13 in Zhou et al., 2023) that contain four predetermined system parameters, including the fast wobble motion of the fluorophore dipole with the half-cone angel *δ* (Forkey et al., 2005). Because this motion is extremely rapid, a larger *δ* would be manifested as a lower ratio between the amplitudes of polarized versus non-polarized signals. A lower ratio would in turn be translated to a smaller apparent *θ* value, which can, however, be corrected with *δ* determined in an ensemble anisotropy study (see Materials and methods) (Forkey et al., 2000; Lewis and Lu, 2019a).

Given that both angles were calculated here from the ratio of intensities, a change in *I*_tot_, due to such a factor as changes in the environment of the fluorophore, would be proportionally reflected in all four polarized components. Consequently, such a change itself would not affect the ratios and thus the determination of the two angles. This approach circumvents the well-known complexity of interpreting the changes in total intensity itself because it depends on the changes in more than one factor, including the aforementioned fluorophore orientation and environment. It is noteworthy that changes in observed *I*_tot_ contain information regarding changes in *θ* but not *φ*, although SNR is too small to deduce the changes in *θ* in the present case.

### Identification of conformational states and analysis of their probabilities

The information encoded in both *θ* and *φ* together gives greater resolution of states and confidence in their identification (Fig. 2 A). Operationally, this resolution was performed in a Cartesian system based on the shortest distance principle, in which x, y, and z coordinates were calculated from *θ* and *φ*, with a unity radius *r* that encodes no meaningful information (Fig. 2 B). The operation was not guided by any preconceived kinetic model with a specific number of states. When two consecutive events were determined to belong to the same state distribution, the transition between them identified by the changepoint algorithm would be considered as a false positive, an example of which is shown in Fig. S1 (orange arrow). These events would then be merged to form a single event.

As previously described for the data collected with or without Arg^+^, the highest number of resolvable states from the data obtained here with Agm^2+^ was also four (Figs. 2 and 3), while maintaining the resolution defined by 2.5σ (Zhou et al., 2023). For communication, we will refer to these states as conformational states *C*_1_–*C*_4_ to distinguish them from structural states, a term reserved for those determined by a structural biology method. As expected, the probability but not the spatial orientation of each state varied with the ligand concentration (Figs. 3 and S2) (Zhou et al., 2023).

The spatial orientations of the fluorophore dipole and thus the tracked helix in *C*_1_ and *C*_4_ were chosen to define the local frame of reference. For all molecules, the local x axis is defined by the orientation in *C*_1_ such that the mean *φ*_1_ = 0°, and the local x-y plane by those in *C*_1_ and *C*_4_ such that the mean *θ*_1_ or *θ*_4_ = 90°. As such, the local coordinates x, y, and z axes, and thus *θ* and *φ* are solely specified by these inherent spatial features of the AdiC protein itself. In this local coordinate system common to all examined molecules, we built the *θ* or *φ* distribution for individual conformational states of these molecules to determine their statistics and the state probabilities (Fig. 3). All four resolved states exhibited meaningful probabilities without or with Agm^2+^, characteristics requiring a model of four apo and four ligand-bound states (*C*_*i*_ and ^L^*C*_*i*_) (Figs. 3 and 4). When both Arg^+^ and Agm^2+^ are considered, 12 states are required. As with Arg^*+*^ (Zhou et al., 2023), the probabilities of *C*_2_ and *C*_3_ clearly vary with the Agm^2+^ concentration, which should thus be open states) (Fig. 4 A). In contrast, the probabilities of *C*_1_ and *C*_4_ displayed relatively small variations, which may thus not be directly accessible to ligands and be consistent with occluded states. Regarding sidedness, judging from available AdiC’s structures alone, the orientations of *C*_1_ and *C*_2_ are consistent with those of E_x_ and E_o_ (Table 1). Based on these two sets of information, the relations of *C*_1_–*C*_4_ to the structural–functional states are consistent with the following assignment: *C*_1_ corresponds to E_x_, *C*_2_ to E_o_, *C*_3_ to I_o_, and *C*_4_ to I_x_, a relation observed also in our previous study of AdiC with Arg^+^ (Zhou et al., 2023).

**Table 1.** Orientations and relative angle changes of the four conformational states of AdiC

	θ_1_ E_x_	θ_2_ E_o_	θ_3_ I_o_	θ_4_ I_x_
Crystallography^a^	93.8°	83.6°	71.7°	95.3°
Polarization (BF^b^, Agm^2+^)	90° ± 5.3°	88.7° ± 6.7°	77.4° ± 9.3°	90° ± 5.3°
Polarization (BF^b^, Arg^+^)	90° ± 4.7°	87.8° ± 7.2°	76.6° ± 9.0°	90° ± 4.5°
Polarization (MF^c^, Arg^+^)	90° ± 4.6°	89.0° ± 5.5°	79.9° ± 7.3°	90° ± 4.1°
	**φ**_**1**_ ** E**_**x**_	**φ**_**2**_ ** E**_**o**_	**φ**_**3**_ ** I**_**o**_	**φ**_**4**_ ** I**_**x**_
Crystallography^a^	−1.89°	20.9°	27.5°	38.7°
Polarization (BF^b^, Agm^2+^)	0° ± 4.5°	17.9° ± 6.3°	27.2° ± 9.3°	40.5° ± 10°
Polarization (BF^b^, Arg^+^)	0° ± 4.0°	16.6° ± 7.2°	26.6° ± 10°	38.5° ± 11°
Polarization (MF^c^, Arg^+^)	0° ± 3.9°	13.7° ± 4.8°	22.7° ± 6.7°	39.5° ± 7.5°

a In the structural analysis, the AdiC structures in the states E_O_ (PDB: 7O82) and E_X_ (PDB: 3L1L) and the BasC and ApcT structures in the states I_O_ (PDB: 6F2G) and I_X_ (PDB: 3GIA) were used. Data are shown as mean ± σ.

b “BF” indicates the fluorophore rhodamine bifunctionally attached.

c “MF” indicates the fluorophore ATTO-550 monofunctionally attached.

We note the following finding revealed originally in that study (Zhou et al., 2023) and observed again here (Fig. S3). The above corresponding relations also turn out in the best fit between the mean orientations of the tracked helix in the four conformational states and those of the crystal structures of AdiC in E_o_ and E_x_, the BasC transporter in I_o_, and the ApcT transporter in I_x_ (Fig. 1, D and E) (Shaffer et al., 2009; Gao et al., 2010; Errasti-Murugarren et al., 2019; Ilgü et al., 2021). These latter two transporters share the same structural fold with AdiC. Among the two sets of four states, there are 24 possible combinations. The best fit combination was determined according to the largest inverse value of combined least squares (*LS*_*e*_) among the 24 cases (Fig. S3 C; right arrow). We will evaluate the transport properties of AdiC predicted by the model in this configuration and compare them with those of the configuration with the opposite membrane sidedness, i.e., *C*_2_ corresponds to I_O_ and *C*_3_ corresponds to E_O_ (Fig. S3 C; left arrow).

Based on the above information, we generated the temporal template for Video 1, with which we show how to exhibit the conformational changes of a single subunit by matching the states identified here at each time point to their corresponding structural states. Because the structures of AdiC in only E_o_ and E_x_ are currently available, the structures of the BasC and ApcT transporters in I_o_ and I_x_ states are used as temporary proxies for those of AdiC (Shaffer et al., 2009; Gao et al., 2010; Errasti-Murugarren et al., 2019; Ilgü et al., 2021). Specifically, the temporal template was obtained from the *Ω* trace in which the states had been identified (Fig. 1 B). The structural states are shown in the form of electron density maps (PDBs: 7O82, 3L1L, 3GIA, and 6F2G) (Shaffer et al., 2009; Gao et al., 2010; Errasti-Murugarren et al., 2019; Ilgü et al., 2021), color coded for states as in the *Ω* trace. Two segments of video frames from the video are shown in Fig. 1 C. Thus, the video was directly generated from the experimental data without kinetic and structural modeling, showing the spatiotemporal behaviors of a single subunit. Note that due to the limit of allowable file size, the video was made for viewing in a small window.

**Video 1.** **A composite video exhibiting intensities of polarized emission of a fluorophore attached to an AdiC protein molecule shown in** Fig. 1 **, integrated intensity values, calculated angles, and electron densities of AdiC in different conformations.** Shown on the left are the original recorded intensities *I*_0_, *I*_45_, *I*_90_, and *I*_135_ displayed from top to bottom; on the middle left are running traces of integrated intensity values with background subtracted; on the middle right are running traces of angles *θ* and *φ* along with *Ω*, where each event is color coded according to the state that AdiC adopts; *C*_1_ (yellow) corresponds to the E_X_ conformation, *C*_2_ (blue) to E_O_, *C*_3_ (orange) to I_O_, and *C*_4_ (cyan) to I_X_; on the right is a video of the AdiC molecule transitioning among four conformational states represented by the electron densities of AdiC (PDBs 7O82 and 3L1L) and those of the related BasC and ApcT transporters proper (PDBs 6F2G and 3GIA), as described in Results. At a given time point, the electron density corresponding to the conformation identified by the polarization study is displayed. Due to the limit of allowable file size, the video was made to be viewed in a small window of such software as Windows Media Player.

### Construction and analysis of dwell-time distributions of conformational states

Detection of transition points and identification of conformational states enabled us to measure the dwell times of an AdiC molecule in individual conformational states. Fig. 5 exhibits the dwell-time distributions with or without a saturating concentration of Arg^+^ or Agm^2+^. To analyze the distributions, we used an exponential function derived for data recorded with a camera at a constant frame rate (Lewis et al., 2017) (Eq. S1), which also addresses the problem arising from missing short events. Unlike those of MthK’s regulatory module (Lewis and Lu, 2019c), the dwell-time distributions of AdiC’s four states under all examined conditions are statistically much better fitted with a double-exponential function than a single-exponential function (Eq. S1 versus Eq. S2), based on all P values being <10^−15^ in F-tests (Woody et al., 2016). The distributions could not be fitted with a triple-exponential function without arbitrarily constraining the fitting parameters of some components. In the final analysis of each state, a global double-exponential fit to the entire collection of distributions of dwell times obtained in the presence of all tested concentrations of Arg^+^ and Agm^2+^. As a requirement to achieve the joint fit, the fast and slow components are each coupled to a binding isotherm that informs the concentration dependence of relative amplitude of a given component. Certainly, the fits to data looked better when each distribution was separately fitted because in such an operation, the statistical noise among different experiments to obtain information under different substrate conditions were not taken into consideration.

**Figure 6.**
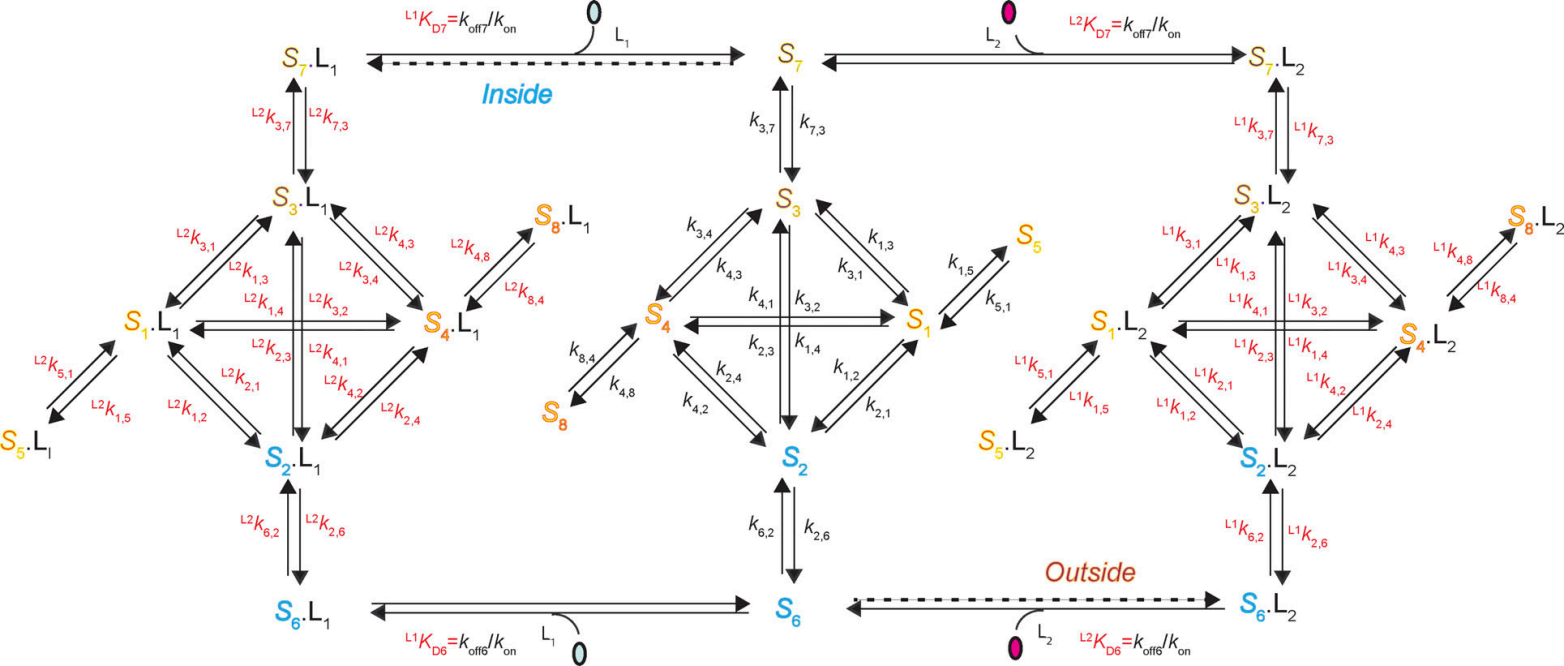
**A 24-state model of AdiC’s conformational kinetics.** All states are denoted as described in Results. The middle portion illustrates the transitions of apo AdiC among eight identified states differing in conformation or energy. The left and right portions illustrate the transitions of AdiC bound with two types of ligand, one side for each type. Transition rate constants *k*_i,j_ and *k*_j,i_, associated with arrows, indicate the reversible transitions between corresponding states *i* and *j* (Tables S1, S2, S3, and S4). The transitions directly involving the binding and unbinding of a ligand are labeled with a $K_{D}=k_{\text{off}}/k_{\text{on}}$.

Given that each of the four conformational states has two identifiable, energetically distinct states, 8 states are required to account for the behaviors of AdiC in the absence or the presence of either ligand type, totaling 24 states (Fig. 6). To reflect this expansion, each of the four conformations in Fig. 4 B needs to be split into two energetic states. The two energetic states of *C*_1_ are denoted as *S*_1_ and *S*_5_, *C*_2_ as *S*_2_ and *S*_6_, *C*_3_ as *S*_3_ and *S*_7_, and *C*_4_ as *S*_4_ and *S*_8_.

The state transition information (Fig. 1), which is necessary for identifying state connectivity, was observed at the level of conformation states, not their underlying energetic states. Thus, the only constrainable model is the one in which one of the two interconnected energetic states of a given conformation directly communicates with one of the two interconnected energetic states of another conformation, dubbed a serial connection (Fig. 6). When the connectivity among states is considered, there are no other constrainable alternative 24-state models. The building of a serial relation for *S*_2_ and *S*_6_ or *S*_3_ and *S*_7_, in terms of accessing a third state or the solution, is consistent with the following mechanistic expectation. A ligand-binding process involves at least two steps: an initial second-order reaction step to form a so-called collision complex between the ligand and its receptor, which is typically a near diffusion-limited process, and a subsequent first-order transition to a more stable bound state. In a serial arrangement, *S*_5_ or *S*_8_ does not directly transition to any open states and is effectively an occluded “cul-de-sac” state (see below). Here, the pair of *S*_1_ and *S*_5_ could be reduced back to *C*_1_, and *S*_4_ and *S*_8_ to *C*_4_ but are separately expressed to reflect the kinetic evidence of their existence.

### Determination of rate constants in the framework of the 24-state model

**Figure 7.**
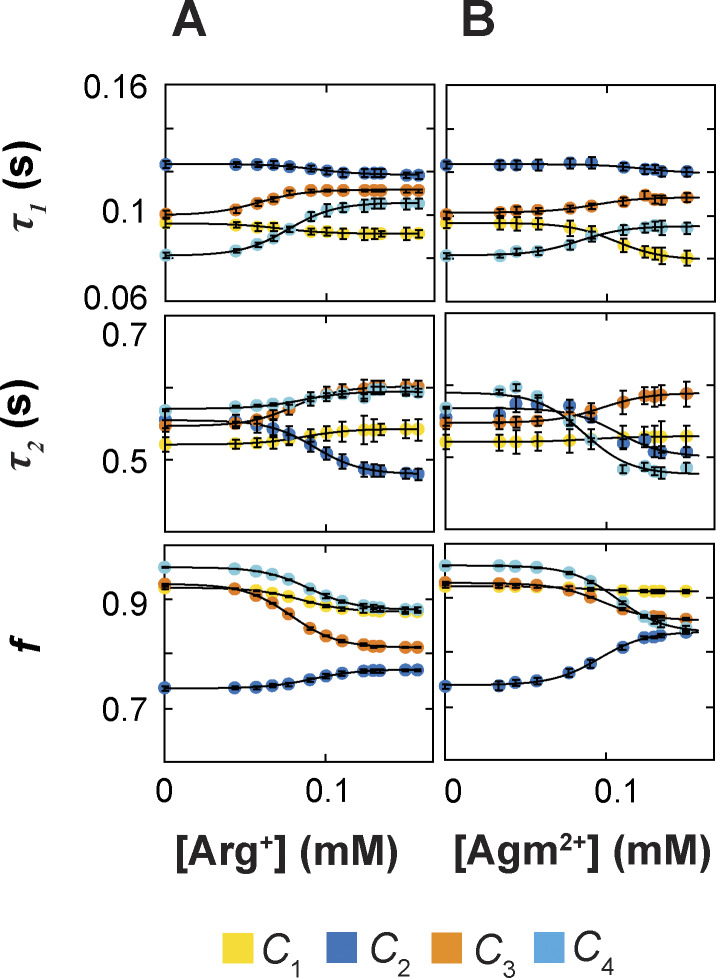
**Dependence of exponential fitting parameters on the concentration of ligands. (A and B)** Time constants *τ*_1_ and *τ*_2_ of the double-exponential components and the relative amplitude *f* of the first component plotted against the concentration of Arg^+^ (A) or Agm^2+^ (B). All parameters were obtained through double-exponential fits to dwell-time distributions as shown in Fig. 5. All data are presented as mean ± SEM, and their representing symbols are color coded for states. The curves superimposed on the data are fits of an equation in the same form as Eq. 3. Fitted parameter values are given in Tables S1, S2, S3, and S4.

**Figure 8.**
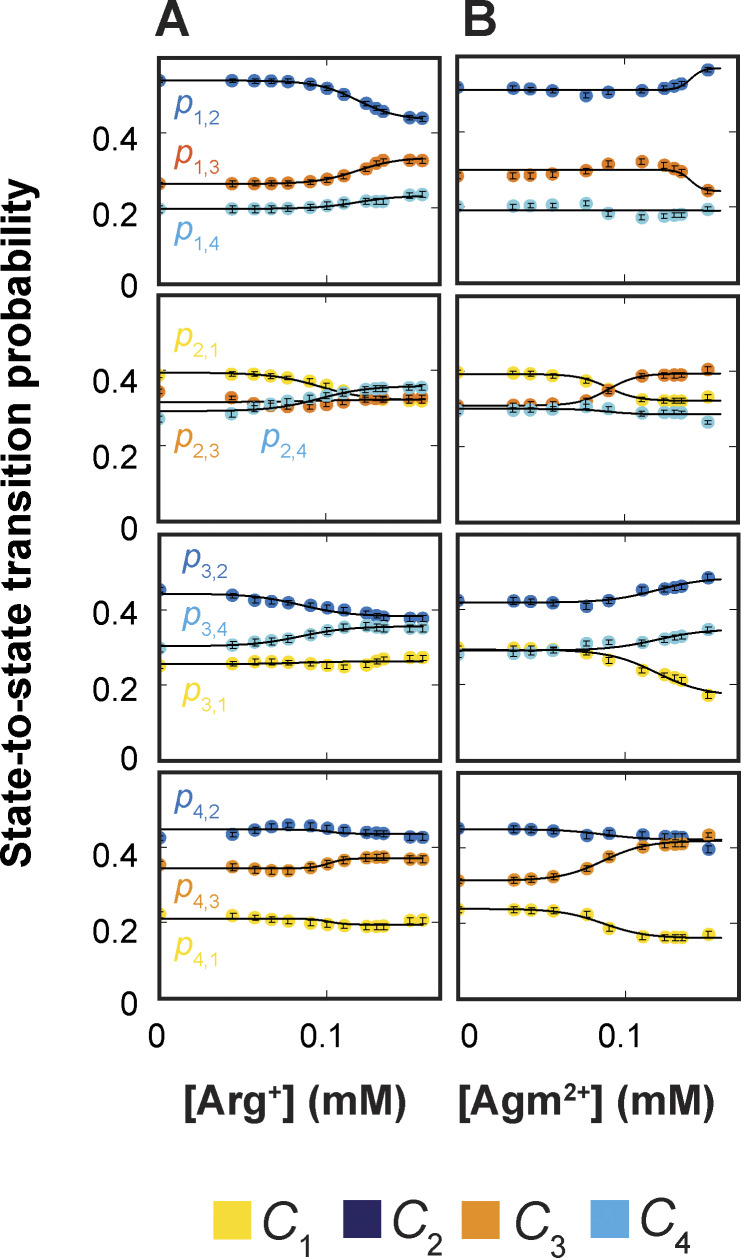
**Dependence of probabilities of state-to-state transitions on the concentration of ligands. (A and B)** Probabilities of state-to-state transitions plotted against the concentration of Arg^+^ (A) or Agm^2+^ (B). All data are presented as mean ± SEM, and their representing symbols are color coded for states. The lines superimposed on the data correspond to fits of the respective groups of data to an equation in the same form as Eq. 3. Fitted parameter values are given in Tables S1, S2, S3, and S4.

**Figure 9.**
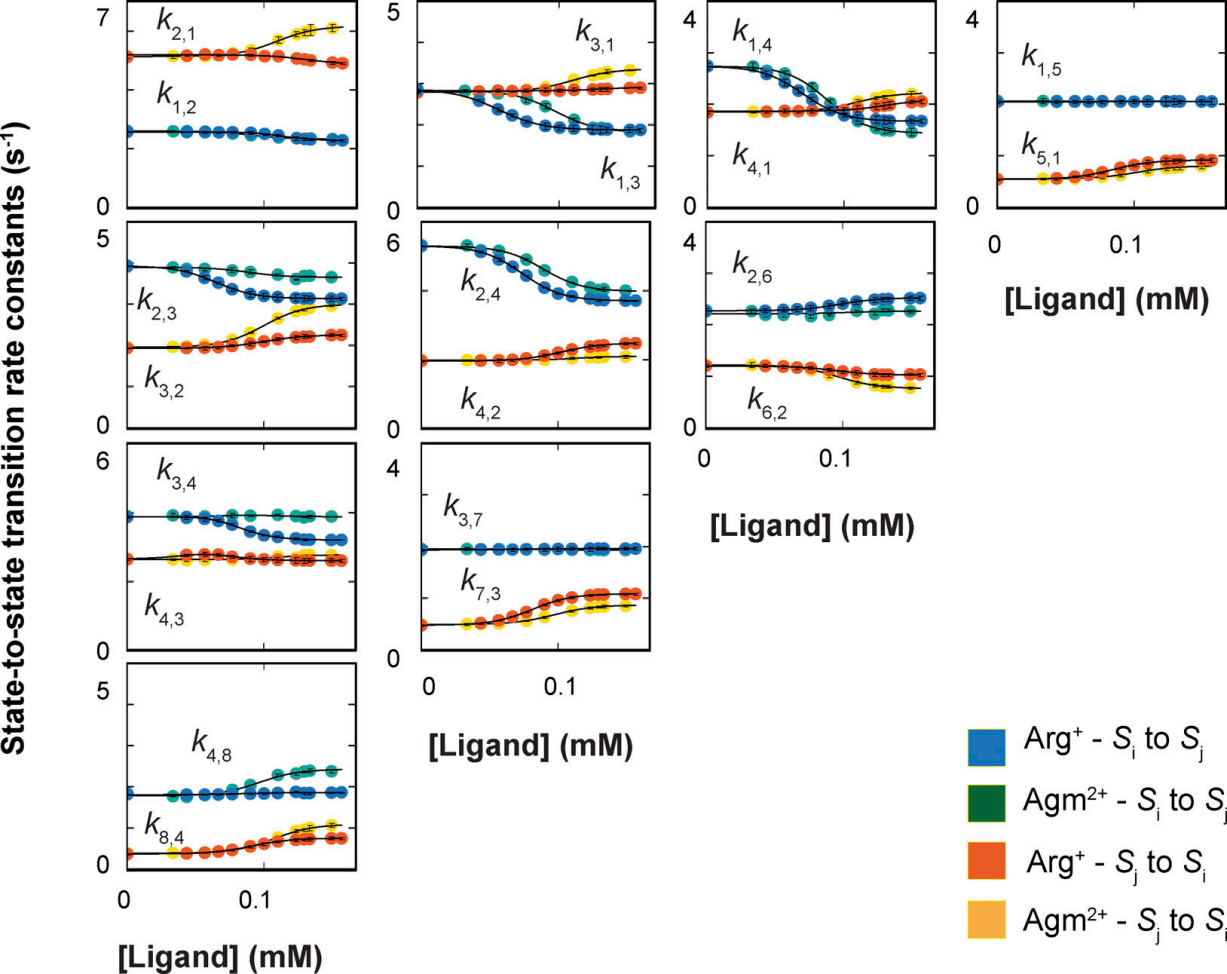
**Dependence of apparent state-to-state transition rate constants on the concentration of ligands.** Apparent rate constants plotted against the concentration of Arg^+^ or Agm^2+^, obtained as described in Results. All data are presented as mean ± SEM. The symbols for (forward) or (backward) rate constants, *k*_i,j_ or *k*_j,i_, are colored blue or red in the case of Arg^+^, whereas they are colored green or orange in the case of Agm^2+^. The curves superimposed on the data are fits of an equation with the same form as Eq. 3. Fitted parameter values are given in Tables S1, S2, S3, and S4.

The analysis of the dwell-time distribution of each confirmation for a given ligand condition yields three parameters: the time constants of the first (fast) and the second (slow) components (*τ*_1_ = 1/*λ*_1_ and *τ*_2_ = 1/*λ*_2_) and the fraction (*f*) of the amplitude of the first exponential component relative to the total. From these parameters and the state-to-state transition probabilities (*p*_i,j_) obtained under the corresponding condition, we could extract the underlying rate constants among all relevant states (see supplemental text at the end of the PDF). In such a way, we performed the analysis on data collected in the presence of 13 Arg^+^ concentrations or 11 Agm^2+^ concentrations, including the ligand-free condition. The values of *τ*_1_, *τ*_2_, and *f* obtained for all examined ligand conditions are plotted in Fig. 7, and those of *p*_i,j_ in Fig. 8. From these parameters of each conformation, we calculated the 20 apparent rate constants ^app^*k*_i,j_ for a given ligand condition and plotted them against the Arg^+^ or Agm^2+^ concentration (Fig. 9). Fitting the equation

kappi,j=ki,j+kLi,j[L]K1/21+[L]K1/2 (3)

to all of the plots in Fig. 9, we obtained the rate constants for apo (*k*_i,j_), Arg^+^-bound, or Agm^2+^-bound AdiC (^L^*k*_i,j_), all summarized in Tables S1, S2, S3, and S4. The fits to the Arg^+^ and Agm^2+^ data were coupled to obtain a single set of rate constants for the apo condition.

The first-order rate constant *k*_i,j_ or ^L^*k*_i,j_ corresponds to the mean rate of a molecule to traverse state *i* to reach state *j*, without being scaled by *p*_i_. A straightforward way to obtain *k*_i,j_ and ^L^*k*_i,j_ is to collect the data under the condition that both sides of AdiC are exposed to the same series of substrate concentrations such that *k*_i,j_ and ^L^*k*_i,j_ can be estimated by extrapolating to the zero and saturating-ligand concentrations, respectively. Here, satisfying this condition, both sides of individual AdiC molecules, each in a nanodisc, faced the same series of solutions. Under the apo, Arg^+^-bound, or Agm^2+^-bound condition, *k*_1,5_, *k*_2,6_, *k*_3,7_, or *k*_4,8_ are markedly slower than the other rate constants, seen in Fig. S4 as taller columns that represent the inverse values of rate constants. Among them, *k*_2,6_ and *k*_3,7_ quantitatively define the transitions on the transport pathway, each of which immediately precedes an actual substrate-releasing step with a much greater rate constant.

### Verification of the observed conformational behaviors with a monofunctional fluorophore

Using monofunctional ATTO-550, we tested whether a fluorophore’s characteristics affect AdiC’s conformational behaviors (Fig. S5). Hydrophobic ATTO-550 is expected to pack itself against hydrophobic local protein elements, but not necessarily along helix 6A. Thus, even if the bifunctional label incidentally affected the conformational changes, a monofunctional label might not act similarly. Without additional information, the spatial relation between helix 6A and ATTO-550 is unknown. However, if their relation remains statistically constant, the relative orientations of ATTO-550 among the states should report those of helix 6A defined by the local coordinates.

For all four resolved states, the angles calculated from the intensities of the mono- and bifunctional fluorophores are comparable (Table 1), after corrected for their wobble angles (39.4° versus 27.0°; Material and methods). The mean σ for *θ* and *φ* of the monofunctional fluorophore are 3.65° ± 2.62° and 3.64° ± 2.56°, compared with 3.73° ± 2.27° and 3.85° ± 2.48° of bifunctional rhodamine. Under the same substrate conditions, the observed probabilities and kinetics of the corresponding states were also comparable for the two compared fluorophore types (Tables S5, S6, S7, S8, and S9).

### Determination of the equilibrium constants among the energetics states

**Figure 10.**
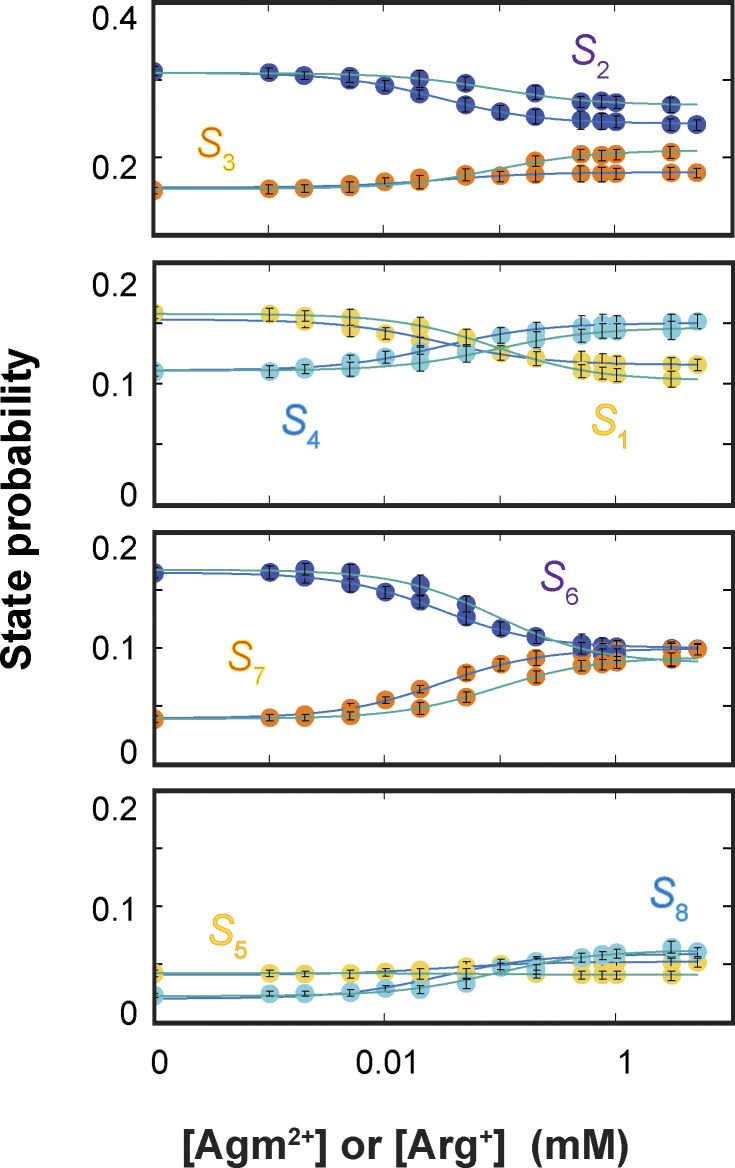
**Dependence of probabilities of energetic states on the concentration of ligands.** Probabilities of states *S*_1_ − *S*_8_ (yellow for *S*_1_ and *S*_5_; blue for *S*_2_ and *S*_6_; orange for *S*_3_ and *S*_7_; and cyan for *S*_4_ and *S*_8_) are plotted against the concentration of Arg^+^ (with fitted curves in blue) or Agm^2+^ (with fitted curves in green). All data are presented as mean ± SEM. The probability values are calculated using Eq. S31 from the rate constants plotted in Fig. 9. The values for the apo and ligand-bound states are given in Table S10. The curves superimposed on the data are fits of Eq. 4. Fitted parameter values for equilibrium dissociation constants are given in Table S11, and the remaining equilibrium constants in Table S12.

To determine all K_D_ values, we first used Eq. S31 to calculate the probabilities of the eight energetic states *S*_1_–*S*_8_ (Fig. 10) from the corresponding ^app^*k*_i,j_ values (Fig. 9) for each ligand condition. The resulting values of state probabilities of apo and Arg^+^- or Agm^2+^-bound states are given in Table S10. The *K*_*D*_ values for individual states (Table S11) and those of other equilibrium constants for apo and Arg^+^- or Agm^2+^-bound states (Table S12) were then determined by a global fit to the eight plots for Arg^+^ or Agm^2+^ in Fig. 10 with the following equation:

Pi=Ki,1+KLi,1[L]KD1∑i=18Ki,1+∑i=18LKi,1[L]KD1 (4)

where *S*_1_ is used as a reference for other states (*S*_i_) to define equilibrium constants:

Ki,1=[Si][S1]; Ki,1L=[Si.L][S1.L]; KDi=[Si][L][Si.L]; i=1,2…8 (5)

The probabilities of *S*_6_ and *S*_7_ exhibit greater variations with varying ligand concentrations than those of other states, characteristics consistent with *S*_6_ and *S*_7_ being the open states. Thus, K_D_ for *S*_6_ or *S*_7_, which characterizes the binding of external or internal ligand Arg^+^ or Agm^2+^, is practically consequential, four of which and the 60 rate constants fully determine the 24-state conformation-kinetic model of AdiC (Fig. 6).

Furthermore, we calculated the relations between the probabilities of *C*_1_–*C*_4_ and the ligand concentration, each from the corresponding pair of curves fitted to the relevant relations of *S*_1_–*S*_8_ in Fig. 10, and overlaid them on the observed relations in Fig. 4 A. As expected, the probabilities predicted for the four conformations from the eight energetic states identified by the kinetic analysis match the observed probabilities over the tested ligand concentration range.

### Monte Carlo simulation of the time courses of conformational state dynamics

**Figure 11.**
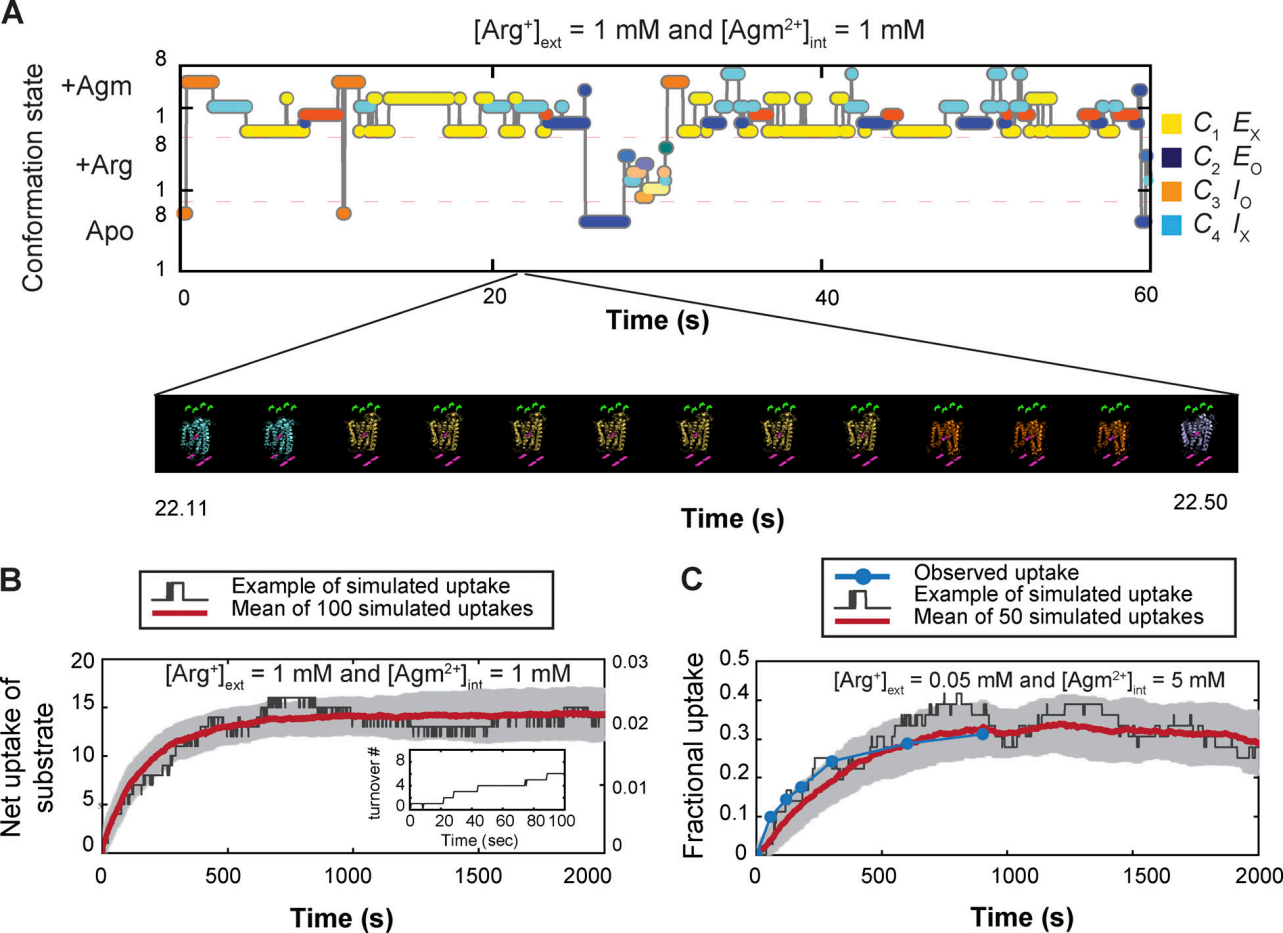
**Example of a simulated time course of conformational transitions of AdiC, a segment of a video illustrating a transporter model in 4D, and simulated time courses of net ligand transport. (A)** Time course of AdiC transitioning among four types of conformations, simulated with the model shown in Fig. S6 as described in Results for the initial presence of 1 mM extracellular Arg^+^ and 1 mM intracellular Agm^2+^. For distinction, AdiC’s presence in the apo, Arg^+^-bound, and Agm^2+^-bound forms are indicated by individual filled circles in the lower, middle, and upper sections of the panel, respectively; its adoptions of *C*_1_ (*S*_1_ + *S*_5_) are colored yellow, *C*_2_ (*S*_2_ and *S*_6_) colored blue, *C*_3_ (*S*_3_ and *S*_7_) colored orange, and *C*_4_ (*S*_4_ and *S*_8_) colored cyan. Shown underneath this simulated time course is a segment from Video 2, exhibiting a 4D model of the transporter proper transitioning among four types of conformations while transporting ligands. The video was generated using the simulated time course shown in the panel A as the temporal template to connect the structures of different states, as described in Results. The shown video frames correspond to the segment of simulated time course from 2,211 to 2,250 ms. **(B)** The time course of uptake of Arg^+^ extracted from the simulation shown in panel A. The black trace illustrates the time course yielded from a simulation of a single subunit of AdiC, where the inset exhibits its first 100 s in an enlarged view. The red trace corresponds to the average of the outcomes from 100 such simulations. **(C)** The observed time course of the fractional net uptake of radioactive Arg^+^ for the initial conditions of 50 μM external radioactive Arg^+^ and 5 mM internal Agm^2+^ (closed blue circles) (Tsai et al., 2012), overlaid with a single simulated time course (black trace) and the mean of 50 such simulations (red trace). The gray colored zone in panels B or C represents what is defined by mean ± σ, corresponding to the 68% confidence interval.

Fig. 11 A exhibits a simulated time course of conformational changes, a type of temporal template used to generate an integrative 4D model of AdiC (Video 2) or to simulate the time-dependent substrate transport (Fig. 11 B). The initial simulated state was designated as *S*_6,_ transitioning to one of the three connected states: *S*_2_, Arg^+^-bound *S*_6_ or Agm^2+^-bound *S*_6_ after a simulated time (*t*_sim_) (Fig. 6). The *t*_sim_ was obtained by randomly drawing from an exponential distribution of dwell times for *S*_6_, defined by the rates of exiting to the three states. When the accumulated time became equal to or longer than *t*_sim_, AdiC would transition from *S*_6_ to one of the three connected states, which was determined by the outcome of a random draw from a multinomial distribution defined by the three state-to-state transition probabilities (Fig. 8). The *t*_sim_ of this second state and its termination were determined as described above. These steps were repeated until the end of the simulation.

**Video 2.** **Video of simulation of a counter transport of Arg** ^**+**^ **and Agm** ^**2+**^ **by AdiC.** As described in Results, at a given time point, the crystal structure model for a specific state is displayed according to the template shown in Fig. 11 A. The *C*_1_ (yellow) state corresponds to the E_X_ conformation, *C*_2_ (light blue) to E_O_, *C*_3_ (orange) to I_O_, and *C*_4_ (cyan) to I_X_. The structures in E_O_ and E_X_ are those of AdiC (PDBs 7O82 and 3L1L), whereas I_O_ and I_X_ are those of the related BasC and ApcT transporters proper (PDBs 6F2G and 3GIA). Arg^+^ (shown in green) is transported from the external (top) side to the intracellular (bottom) side, whereas Agm^2+^ (shown in maroon) is transported in the opposite direction. Throughout the video, external Arg^+^ and internal Agm^2+^ are both kept at constant 1 mM. The number of transported substrate molecules is assumed to be negligible with respect to the total number of available substrate molecules. Due to the limit of allowable file size, the video was made to be viewed in a small window of such software as Windows Media Player.

### Characteristics of two alternative 24-state transporter models of opposite sidedness

In the 24 conformational state model, the rate constants of all shown pathways are fully locked in by experimental data. Then, a natural question is whether this model can reasonably predict the observed Michaelis–Menton kinetic behaviors of AdiC, a model analytically expressed in the form of a system of 24 differential equations of no adjustable parameters under a given substrate condition (see supplemental text at the end of the PDF).

The substrate dependence of the rate of Arg^+^ or Agm^2+^ uptake into lipid vesicles embedded with AdiC has previously been examined (Tsai et al., 2012). In their study, the initial concentration of Arg^+^ in the vesicles was always 5 mM, which is a practically saturating concentration, whereas the bathing medium contained a varying initial concentration of radio-labeled Arg^+^ and Agm^2+^. To achieve a directed cysteine-based modification, they replaced native cysteine residues in AdiC, which modestly lowered the apparent transport rate. We also replaced cysteines to avoid off-target labeling. To compare with those functional data, we performed Monte Carlo simulations of the time courses of conformational state dynamics for individually tested conditions (Fig. 11 A), according to the conformation-kinetic model (Fig. 6). In this example, the functionality of conformational states is assigned as shown in Fig. S6. In the simulation, a release of a substrate molecule to the inside occurs during the transition of substate-bound *S*_7_ to the apo *S*_7_, whereas a release of a substrate molecule to the outside occurs during the transition of substrate-bound *S*_6_ to apo *S*_6_. Fig. 11 B exhibits a simulated ∼30 min time course of the net Arg^+^ uptake and the average of those from 100 independent simulations. We also simulated a previously reported ∼30 min time course of the uptake of radioactive Arg^+^ for the initial substrate conditions of 50 μM external radioactive Arg^+^ and 5 mM internal nonradioactive Agm^2+^ (Tsai et al., 2012). This simulated time course mimics the observed one in terms of not only the time constant but also the maximum fractional uptake ($F_{\max}^{obs}$) (Fig. 11 C). Predicting $F_{\max}^{obs}$ by the model with an apo path for the protein-conformational transitions suggests that AdiC is not an exchanger.

**Figure 12.**
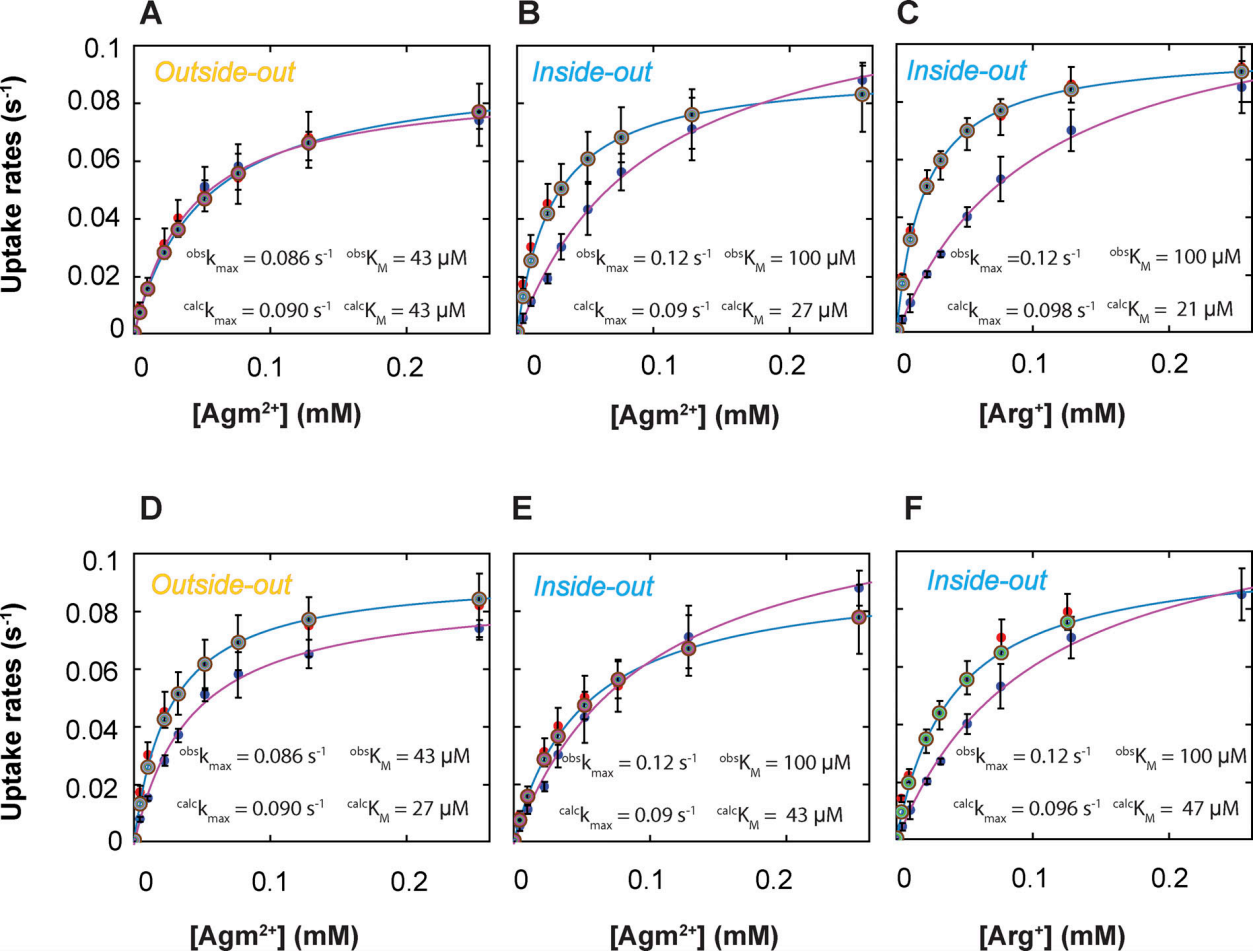
**Comparison of observed and model-predicted rates of AdiC-mediated ligand uptakes into lipid vesicles. (A–F)** The predicted values by the model shown in Fig. S6 (A–C) or those by the model in Fig. S7 (D–F) are compared with corresponding observed values for a given experimental condition (Tsai et al., 2012). Net ligand uptake rates are plotted against the ligand concentration: Agm^2+^ via AdiC in the outside-out (A and D) or inside-out (B and E) orientation and Arg^+^ via AdiC in the inside-out orientation (C and F; see Fig. S9 for the comparison and comments regarding the outside-out orientation). The experimental data are plotted as mean ± SEM, and the predicted values of the alternative models shown in Figs. S6 and S7 by means of Monte Carlo simulations or calculations are plotted as mean ± σ calculated from the corresponding confidence interval. The observed data set is represented by closed dark blue circles, the simulated set of the model by closed red circles, and the five calculated sets, one for each transition, by five of practically concentric open circles. The curves, which are superimposed on the symbols that represent the calculated values, correspond to the fits of the Michaelis–Menten equation. All fit parameters of calculated values are presented in Table S13, along with those reported by Tsai et al. (2012). All calculations were performed as described in the supplemental text at the end of the PDF.

From the observed time course of uptake, Tsai et al. (2012) obtained the relation between the apparent initial uptake rate (^*app*^*k*_flux_) and the substrate concentration (Fig. 12, closed dark blue symbols). The relations predicted by the model where *C*_2_ corresponds to E_o_ and *C*_3_ to I_o_, dubbed sidedness configuration 1, is compared with the observed ones in Fig. 12, A–C (the closed red circles), and those by the model where *C*_2_ corresponds to I_o_ and *C*_3_ to E_o_ (configuration 2) in Fig. 12, D–F. These two models with opposing sidedness are depicted in Figs. S6 and S7. The predicted relations of the two models are statistically comparable with the observed ones. The mean difference between the predicted and observed values is 1.5 or 1.0 times the mean σ for configuration 1 or 2. Consequently, the values of apparent maximum uptake rate (*k*_max_) and the concentrations (*K*_m_) at half of *k*_max_, which empirically characterize the observed and predicted relations, are also comparable (Table S13). The similar predictions of the two models reflect the nearly symmetric transport-kinetic behaviors in the two directions under the examined conditions (Tsai et al., 2012). While Tsai et al. (2012) performed their study under a condition where a “leak” current dissipated the membrane potential caused by a counter transport of Arg^+^ and Agm^2+^ (Fang et al., 2007), we examined AdiC in a nanodisc preparation without membrane potential. Thus, we did not consider the membrane potential in either the above Monte Carlo simulations or the following calculations.

A second way to simulate the model-predicted transport characteristics is through direct calculations, using the solutions to the system of 24 differential equations (Eq. S30). One equation corresponds to one state in the 24-state model where the radioactive substrate would be represented by L_E_ and the nonradioactive substrate by L_I_ (Figs. S6 and S7). This analytic system is constrained by the 60 determined rate constants for the state-to-state transitions in the model plus four *K*_*D*_ values. Operationally, each *K*_*D*_ needs to be split to the underlying on- and off-rate constants (*k*_on_ and *k*_off_). The association and dissociation of substrate are treated as a rapid equilibrium process. All *k*_on_ values set to the near diffusion-limited rate 10^7^ M^−1^ s^−1^, and *k*_off_ values, calculated from the respective *k*_on_*K*_*D*_, are much larger than other 60 rate constants; none of the calculated k_off_ values limits calculated transporting rates. By these calculations, we directly evaluated the relation between ^*app*^*k*_flux_ and each of the five serially related transitions along the transport path in the model. That is, the transition between *S*_7_ and *S*_7_*L*, *S*_7_*L* and *S*_3_*L*, *S*_3_*L* and *S*_2_*L* (whose rate was calculated as the sum of the rates of three underlying parallel pathways along the direction), *S*_2_*L* and *S*_6_*L*, or *S*_6_*L* and *S*_6_. Under a given substrate condition, each rate is calculated as the difference between the forward and backward rates of the transition between, e.g., *S*_*2*_*L* and *S*_6_*L*:

kappflux=kL2,6PL2(L)−kL6,2PL6(L) (6)

For a given condition, the rate was calculated using time-dependent substrate concentrations and state probabilities (see supplemental text at the end of the PDF). As such, both the changes in the concentrations of a given substrate species and the changes in probabilities of individual states in response to the changing substrate concentrations were taken into consideration.

For a given AdiC orientation and specific substrate conditions, the calculated relation between ^*app*^*k*_flux_ and the substrate concentration is the same for the five transitions and very similar to the corresponding one obtained in the above Monte Carlo simulation (Fig. 12; a set of concentric circles versus the corresponding closed red circles). As such, the flux rate across the system in the direction of transport is essentially uniform and can be approximated by that of each of those transitions. Thus, following a perturbation caused by a change in the substrate concentration, the conformation-energetic states of AdiC themselves reach a new (near) equilibrium state within a timeframe much shorter than the process of the net transfer of a single substrate molecule. Such a characteristic is expected for the case where the forward and backward rates of individual transitions are comparable, and the net flux rate reflects the difference between the rates in two opposite directions.

If the flux rate can be estimated from a single transition, one may wonder what the importance of the information regarding all the remaining states is. This importance stems from that the apparent transition rate for a state to another state is given by the product of the rate constant and the state probability, and its variation with concentration solely reflects the ligand-dependent variation of the state probability (Eq. 6). Thus, the flux-rate calculation for a given condition requires knowing the probabilities of the two relevant states under that condition, besides their invariant transition-rate constants. The determination of the probabilities of a pair of states in turn requires knowing the probabilities of all remaining states either individually or together, here estimated for a given set of substrate conditions through calculating the time-dependent probabilities of the 24 states from experimentally determined values of rate constants and *K*_*D*_ (see supplemental text at the end of the PDF). What is outlined above highlights the importance to track all states and points to that the occluded cul-de-sac states *S*_5_ and *S*_8_ indirectly affect the transport rate through their probabilities, acting like sinks.

It is noteworthy that the predicted initial rate of transport in the above calculations or Monte Carlo simulations are the true initial rates of the models, whereas the experimental values tend to underestimate initial rates because of low signal-to-background ratio when the substrate concentrations in bathing solution are low and limited time resolution, the latter of which has been shown by Tsai et al. (2012). This issue should, in part, underlie the model-predicted rates being nearly equal or modestly greater than the observed rates (Fig. 12).

Additionally, because in the model the transport of each substrate molecule is coupled to AdiC’s conformational transitions, the comparability between the model-predicted and observed *k*_max_ values strongly supports the notion that AdiC is a facilitating transporter rather than a channel, as the latter would allow vastly more than one molecule to pass through it during each adoption of an open conformation. This conceptual distinction is arguably of importance here, given that AdiC does not appear to be an obligatory exchanger but a uniporter.

**Figure 13.**
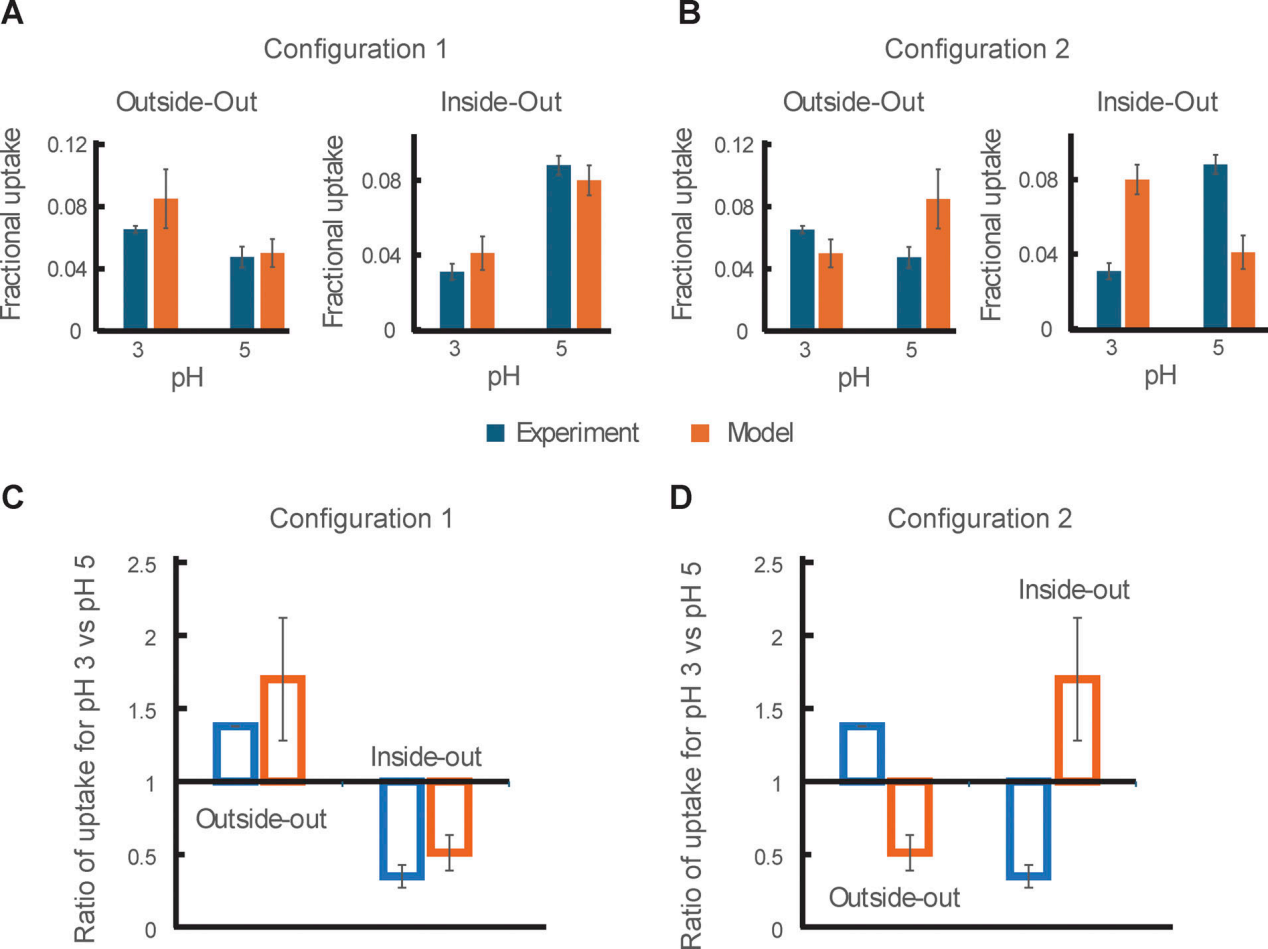
**Simulations of the pH-dependent uptake of Arg** ^**+**^ **under asymmetric conditions using our model in two configurations of opposite sidedness. (A and B)** Fractional uptake values (mean ± SEM) were previously reported by Tsai et al. (2012), which were obtained via assaying radioactive Arg^+^ fluxes into vesicles with AdiC reconstituted in the outside-out or inside-out orientation (closed blue bars). As initial conditions, the solution of pH 5 in vesicles contained 5 mM Arg^+^, whereas the bathing solution of pH 3 or pH 5 contained 50 μM of radioactive Arg^+^. The fractional uptake values were determined 5 min after the initiation of the experiments. We simulated their data using our model in the configurations 1 (A) and 2 (B) of opposite sidedness illustrated in Figs. S6 and S7, respectively (closed oranges bars; mean ± σ). The kinetic parameters for the two different sides of the model were those determined in the corresponding pH conditions. **(C and D)** The ratios between the experimental values for pH 3 and pH 5 in the outside or inside-out orientation (open blue bars) compared with those predicted by the model in configuration 1 (C) and 2 (D) (open orange bars).

Regarding the model’s sidedness, the above comparison of the models of opposing sidedness with the observed transport behaviors has not been informative because of the relative symmetry at symmetric pH of ∼5. However, Tsai et al. (2012) showed that when pH for the extracellular side of AdiC was lowered from 5 to 3, the fractional uptake increased and the ratio of fractional uptakes under these two conditions was less than one. In contrast, when pH for the cytoplasmic side of AdiC was lowered from 5 to 3, the fractional uptake decreased, and the ratio was greater than one. Thus, we further performed the polarization study at pH 3 to determine the individual parameters in our model at this pH. Judging from the opposite effects of lowering pH from 5 to 3, the predictions of the model in configuration 1 but not 2 (Figs. S6 and S7) are consistent with their observations (Fig. 13, A and C versus Fig. 13, B and D).

**Figure 14.**
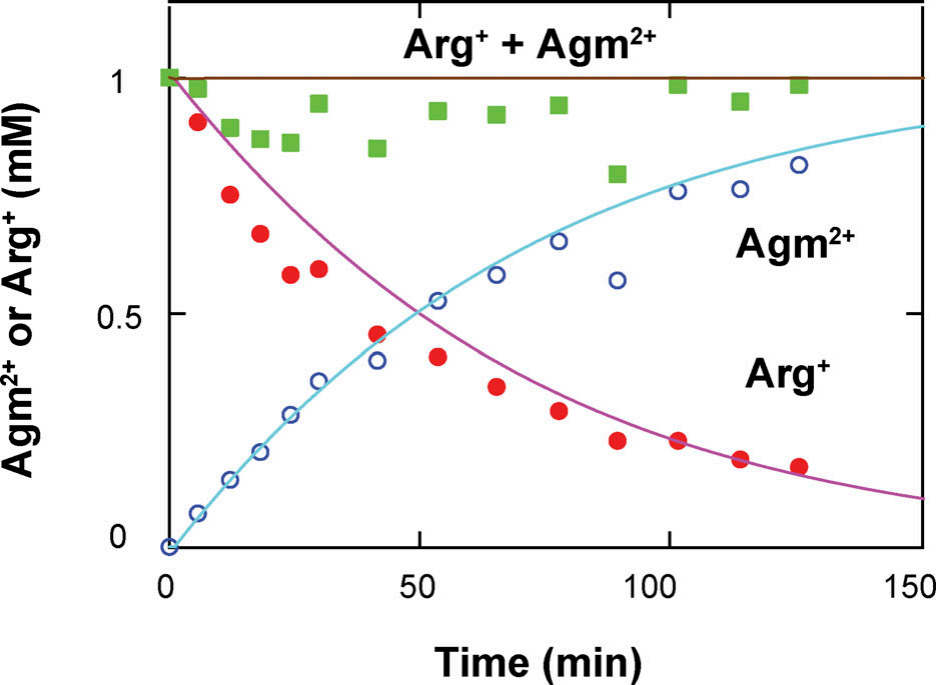
**Comparison of observed and model-predicted rates of substrate transport across bacterial membranes. **The observed concentration of Arg^+^ (red closed circles), Agm^2+^ (blue open circles), or their sum (green squares) in the bacteria culture medium plotted against observation time (Iyer et al., 2002). The curves overlaid on the experimental data represent the calculated Arg^+^ (magenta) and Agm^2+^ (blue) concentrations with the model depicted in Fig. S6. The brown line on top corresponds to the sum of the calculated Arg^+^ and Agm^2+^ concentrations. All calculations were performed as described in the supplemental text at the end of the PDF.

Macroscopically, a 1:1 exchanger transports equal amounts of substrates in opposite directions. The experimentally observed AdiC-mediated Arg^+^ uptake into bacteria was comparable with Agm^2+^ extrusion and, consequently, the sum of Arg^+^ and Agm^2+^ in the medium remained arguably constant over time (Iyer et al., 2002). However, these observations were quantitatively simulated using our model of a uniporter, a model that allows AdiC to indirectly transition between E_o_ and I_o_ even when it is not bound with any substrate (Fig. 14; see supplemental text). The observations likely reflected the experimental conditions of Iyer et al. (2002), under which the substrate concentrations on both sides of the bacterial membrane were sufficiently high such that the probabilities of the apo states were minimized. Thus, the results of Iyer et al. (2002) are necessary but insufficient evidence for AdiC being an obligatory 1:1 exchanger instead of a uniporter.

Furthermore, Iyer et al. (2003) examined the dependence of the Agm^2+^-efflux rate from bacteria on the Arg^+^ concentration in the medium. For clarity, we replotted their observed rate values against the Arg^+^ concentration on a logarithmic scale (Fig. S8). The simulations of our model generally match their data (closed blue circles verses green squares), except for the rate at the highest tested Arg^+^ concentration, which is lower than that at a lesser concentration and thus most likely reflects an experimental error. These simulations show the trajectory expected for a uniporter, i.e., the efflux rate approaches a nonzero minimum as the external substrate concentration is lowered toward zero. However, after eliminating the apo pathway for the conformational changes to mimic an exchanger, the model failed to predict the data of Iyer et al. (closed red circles verses green squares), while exhibiting a key characteristic of exchangers, i.e., the efflux rate approaches zero as the external substrate concentration is lowered toward zero.

### Radioactive substrate flux assays to test the transport model with an apo pathway for conformational transitions

If the observed transitions of AdiC among the apo states are genuine and not caused by such experimental factors as the fluorophore label, unlabeled AdiC should not act as an exchanger. Thus, we examined this prediction using traditional radioactive uptake assays.

The uptake of radioactive substrate via exchangers into vesicles can be rationalized using a simple thermodynamic system of two compartments connected via a 1:1 substrate exchange, without any meaningful interactions with the environment during the considered timeframe. At the time *t* = 0, the internal (*i*) compartment, representing all vesicles together, contains nonradioactive substrate (*NR*) of the concentration [*NR*_*i*_](0), whereas the external (*e*) compartment, representing the external bathing solution, harbors radioactive substrate (*R*) of the concentration [*R*_*e*_](0). A substrate exchange between the two compartments merely allows substrate molecules in them to move and mix. Necessitated by the law of mass conservation, an exchange-based mixing process cannot alter the combined number of substrate molecules or concentration of different substrate types in either compartment. Furthermore, the second law of thermodynamics necessitates the maximization of the system’s entropy when equilibrium is reached at *t* = *∞*, such that the probability (*p*^*R*^) to pick out an *R* from either compartment should be the same:

$$p_{i}^{R}=\frac{n_{i}^{R}\left(\infty \right)}{n_{i}^{R}\left(\infty \right)+n_{i}^{NR}\left(\infty \right)}=\frac{1}{1+\frac{n_{i}^{NR}\left(\infty \right)}{n_{i}^{R}\left(\infty \right)}}$$ (7)

and

$$p_{e}^{R}=\frac{n_{e}^{R}\left(\infty \right)}{n_{e}^{R}\left(\infty \right)+n_{e}^{NR}\left(\infty \right)}=\frac{1}{1+\frac{n_{e}^{NR}\left(\infty \right)}{n_{e}^{R}\left(\infty \right)}}$$ (8)

where *n*^*R*^ or *n*^*NR*^ denotes the number of *R* or *NR*. The equality between Eqs. 7 and 8 leads to

$$\frac{n_{e}^{NR}\left(\infty \right)}{n_{e}^{R}\left(\infty \right)}=\frac{n_{i}^{NR}\left(\infty \right)}{n_{i}^{R}\left(\infty \right)}=\frac{n_{e}^{NR}\left(\infty \right)+n_{i}^{NR}\left(\infty \right)}{n_{e}^{R}\left(\infty \right)+n_{i}^{R}\left(\infty \right)}=\frac{n_{i}^{NR}\left(0\right)}{n_{e}^{R}\left(0\right)}$$ (9)

Under these conditions, the maximal fractional uptake (*F*_*max*_) is expressed as

$$F_{\max}=\frac{n_{i}^{R}\left(\infty \right)}{n_{e}^{R}\left(0\right)}=\frac{1}{1+\frac{n_{e}^{R}\left(0\right)}{n_{i}^{NR}\left(0\right)}}=\frac{1}{1+\frac{\left[R_{e}\right]\left(0\right)V_{e}}{\left[{NR}_{i}\right]\left(0\right)V_{i}}}=\frac{1}{1+\frac{r_{v}}{r_{c}}}$$ (10)

*F*_*max*_ and the first three ratios in Eq. 9 all describe the outcomes of the equilibrating process of radioactive substrate being partitioned between the bathing solution and vesicles via a 1: 1 exchange. Consistent with not only theory but also intuition, these outcomes are predetermined by $\frac{n_{e}^{R}\left(0\right)}{n_{i}^{NR}\left(0\right)}$ or $\frac{r_{v}}{r_{c}}$. Thus, after the accumulation of radioactive substrate in the vesicles reaches the maximum, this maximal level should persist until the substrate condition is altered.

**Figure 15.**
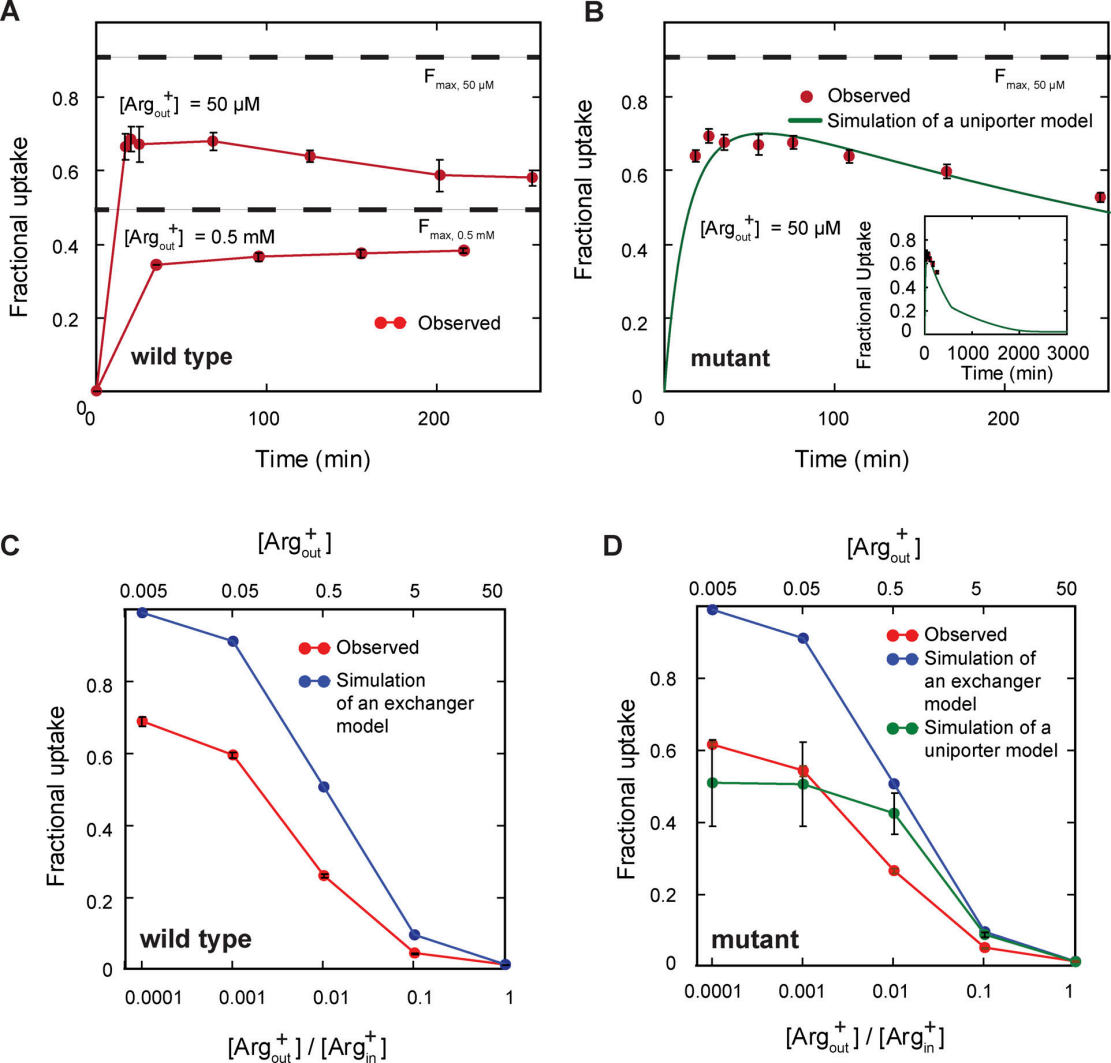
**Radioactive flux assays of wild-type and mutant AdiC. (A and B)** Time courses of radioactive Arg^+^ uptake from the bathing solution into the lipid vesicles embedded with the wild-type (A) and the mutant (B) AdiC molecules. The initial concentration of nonradioactive Arg^+^ inside the vesicles was 50 mM in all cases. At the time *t* = 0, radioactive Arg^+^ at a concentration 0.5 mM or 50 μM in A and 50 μM in B were added to the bathing solution to initiate the transport process. The graph in B is plotted for a >10-fold longer period in the inset. Data points are presented as fractional uptakes (mean ± SEM), defined as radioactive Arg^+^ inside the vesicles as a fraction of the total radioactive Arg^+^ in the sample. The number of assays were 3–6, except 2 for the leftmost data point for the 0.5 mM condition in A. The black dashed lines indicated the theoretically maximum fractional uptake expected for a 1:1 exchanger under the corresponding conditions. The smooth curve (green) overlaid on the data in B is calculated with the model, as described in Results and in Supplemental material. Given the sidedness of AdiC is random in the vesicle, the average of simulations with two models in Figs. S6 and S7 is plotted, labeled as simulation of a uniporter model. **(C and D)** Uptake of radioactive Arg^+^ with different initial Arg^+^ concentrations in the bathing solution while the initial concentration of nonradioactive Arg^+^ in the vesicles was always 50 mM. The fractional uptakes (mean ± SEM; *n* = 3) via the wild-type (C) or the mutant (D) AdiC molecules at *t* = 4 h are represented by red dots, plotted against the concentration of Arg^+^ in the bathing solution (upper x axis) or against the ratio of initial Arg^+^ concentrations in the bathing solution and in the vesicles (lower x axis). The blue dots are the theoretical maximum uptake predicted for a 1:1 exchanger under the corresponding substrate conditions. The green dots in D represent the fractional uptakes (mean ± σ) simulated by means of a Monte Carlo method, using our model with an apo path for conformational transitions, which is labeled as simulation of a uniporter model, as described above. An equal ratio of the two configurations of the model was used in all simulations to mimic the random insertions of AdiC in the two opposite membrane orientations in the flux assays.

To test this prediction for a 1:1 exchanger, we focused on examining the sustainability of the maximum accumulation, over a period of 3 or 4 h following initiation of the uptake process, which was much longer than 2.5–30 min in previous studies (Fang et al., 2007; Tsai et al., 2012; Tsai and Miller, 2013). We first examined the wild-type AdiC protein, allowing the maximal accumulation of radioactive Arg^+^ in the vesicles under the conditions of [*NR*_*i*_](0) = 50 mM and [*R*_*e*_](0) = 0.5 mM. The fractional uptake remained practically constant over 3 h (Fig. 15 A). While this finding is consistent with AdiC being an exchanger, it could also reflect the behavior of a transporter with an apo pathway for conformational changes, such as a uniporter, that acted like an apparent exchanger when the starting substrate concentrations on both sides of the vesicle membranes were much higher than the respective K_D_ values such that the utilization of the apo pathway would be negligible. For a genuine exchanger, the fractional uptake should remain constant over time, even if we repeated the study with a much lower starting substrate concentration of the bathing solution. By contrast, for a uniporter, a drop of the fractional uptake might be observed over an adequately long observation period if the starting substrate concentration were sufficiently low. Thus, we lowered the starting concentration of the bathing solution from 0.5 mM to 50 μM and found that the fractional uptake dropped modestly over 4 h. This drop should not reflect a non-AdiC–mediated leak of vesicles because it only occurred when the bathing solution initially contained a low (50 μM) but not a saturating high concentration (0.5 mM) of Arg^+^. Next, we repeated the study with the mutant AdiC protein used in our polarization study under the conditions of [*NR*_*i*_](0) = 50 mM and [*R*_*e*_](0) = 50 μM, the latter of which is comparable with the observed or model-predicted *K*_m_ values (Figs. 12 and S9). The level of accumulation of radioactive substrate via the mutant AdiC also dropped with time (Fig. 15 B). This drop should primarily reflect a net efflux of both radioactive and nonradioactive substrates along the concentration gradient in the presence of an apo path for the conformational transitions, as the full mixing of radioactive and nonradioactive species would largely be completed near the peak. Our kinetic model of the AdiC transport with all parameters fixed by the polarization data predicts the declining time course observed with the same AdiC mutant used in the polarization study (Fig. 15 B). It also predicts that the maximum drop would occur over >40 h (Fig. 15 B, inset).

Ultimately, we must quantitatively evaluate $F_{\max}^{obs}$ in a thermodynamically predictable manner, analogous to the evaluation of a channel’s ion selectivity. This selectivity is commonly evaluated by comparing the reversal potential on the transmembrane ion-concentration gradient to the prediction of the Nernst equation. Here, to further determine whether AdiC is an obligatory exchanger, we examined $F_{\max}^{obs}$ against expected *F*_*max*_ under various substrate conditions. The equilibrium quantity *F*_*max*_, by means of a 1:1 exchanger, is a function of $\frac{r_{v}}{r_{c}}$ (Eq. 10). Thus, as a prerequisite for calculating *F*_*max*_ at a specific *r*_*c*_, *r*_v_ needs to be determined (Eq. 10). For both exchanges or uniporters, under the symmetric concentration condition of [*NR*_*i*_](0) = [*R*_*e*_](0) or *r*_*c*_ = 1, *r*_v_ can be calculated as

$$r_{v}=\frac{1-F_{\max}^{obs}}{F_{\max}^{obs}}$$ (11)

For the condition of [*NR*_*i*_](0) = [*R*_*e*_](0) = 50 mM, we determined *r*_v_ as 129 ± 8 or 110 ± 7 for wild-type or mutant preparations (see Materials and methods) and used them to calculate the expected *F*_*max*_ values for comparison with those observed in the following studies.

Fig. 15, C and D, shows the relation between the fractional uptake via the wild-type or mutant AdiC at the end of 4 h (*F*_4 *h*_), observed under the conditions that the initial concentration of radioactive Arg^+^ in the bathing solution was varied over a 5 log-unit range, whereas the initial concentration of nonradioactive Arg^+^ in vesicles was always 50 mM. As expected, the fractional uptake was higher when the starting Arg^+^ concentration in the bathing solution was lower. If AdiC were an exchanger and could therefore maintain the *F*_*max*_, *F*_4 *h*_ should match the predicted *F*_*max*_ for a given condition (Eq. 10). However, *F*_4 *h*_ diverges from the predicted *F*_*max*_ as the Arg^+^ concentration in the bathing solution was lowered, a behavior incompatible with an exchanger but a uniporter. Our model with the parameters fixed by the polarization data obtained with an AdiC mutant agrees with the radioactive flux data of the same mutant (Fig. 15 D).

A similar phenomenon was found by analyzing data of Fang et al. (2007). In their experiments, $F_{\max}^{obs}$ was about 0.5 or 0.3 under the conditions that the estimated *r*_v_ was about 50; the initial radioactive Arg^+^ in the bathing solution was 50 µM and nonradioactive Agm^2+^ or Arg^+^ in the vesicles was 5 mM; a leak current dissipated the membrane potential. The $F_{\max}^{obs}$ value for the Agm^2+^ or Arg^+^ condition is indicated by the blue or green line in Fig. S10 A, both of which are markedly below the theoretical *F*_*max*_ of 0.67 (black dashed line). By contrast, our model-predicted *F*_*max*_ reasonably agrees with $F_{\max}^{obs}$ with about 1σ separation; closed blue or green circles represent the simulated values for the presence of 5 mM Arg^+^ or Agm^2+^ in the vesicles. Furthermore, after eliminating the apo path for conformational changes in our model, the resulting exchanger model predicted the fractional uptake to approach the theoretical *F*_*max*_ (Fig. S10 B, open black and maroon open squares versus the dashed line). Additionally, our model also predicts a substrate-dependent efflux from vesicles (Fig. S11 A), a phenomenon previously shown by Fang et al. (2007).

**Figure 16.**
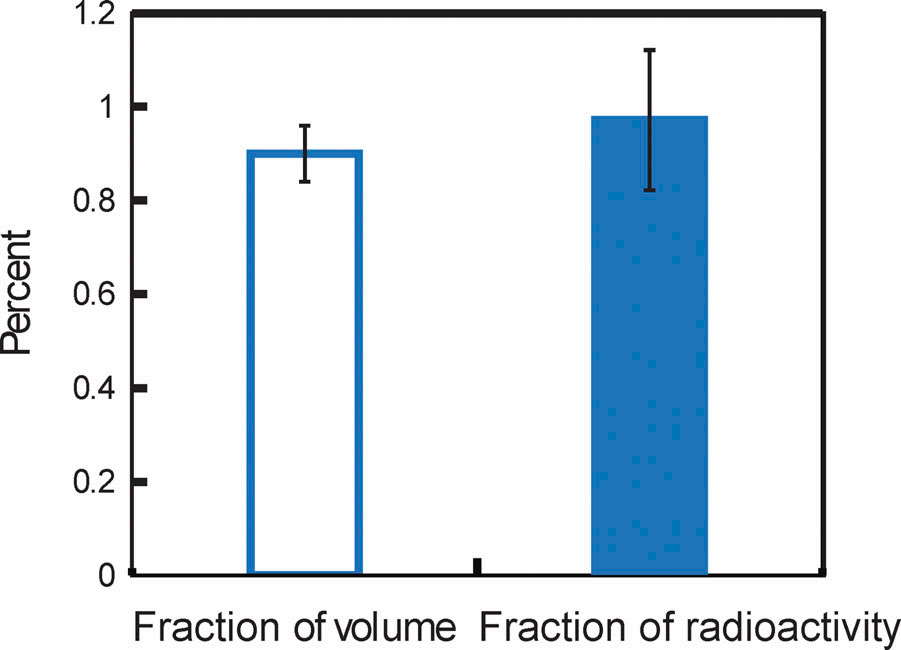
**Comparison between the fractions of volume and radioactivity of vesicles.** The fraction of volume of vesicles as a percentage of the total reaction volume (mean ± SEM; *n* = 3) and that of radioactivity in the vesicles as a percentage of total radioactivity (*n* = 6) determined 2.5 h after initiation of the experiment are plotted as an open bar and a closed bar, respectively. At the beginning of experiment, the vesicles were free of substrate, whereas the bathing solution contained 2 mM radioactive Arg^+^.

Absent any transportable substrate in the vesicles, exchangers cause no uptake beyond their single turnover. By comparison, for a uniporter, radioactive Arg^+^ should be partitioned between the bathing solution and vesicles simply according to their volume ratio at equilibrium. The fractional uptake would be given by $\frac{1}{1+r_{v}}$ because the concentrations of radioactive Arg^+^ should become the same on both sides of the membrane. Calculated from *r*_v_ = 110 ± 7 of the mutant AdiC, the fractional volume of all vesicles was 0.90% ± 0.06%. This fractional volume statistically matches the fractional uptake of 0.97% ± 0.15% (*n* = 6) at 2.5 h from the start of the assay when the vesicle contained no substrate, a match expected for a uniporter (Fig. 16). Similarly, Fang et al. (2007) reported an ∼0.02 fractional uptake at ∼30 min when the vesicle contained nonfunctional D-Arg^+^ (the black line in Fig. S10 A). This fractional uptake of ∼0.02 (above the control taken as being obtained using AdiC-free vesicles) also matches the estimated vesicles’ fractional volume of ∼0.02. Our uniporter model predicts a fractional uptake of 0.02 under their conditions (closed black diamonds in Fig. S10 A).

Additional evidence contradicting the exchanger hypothesis is an increase in $F_{\max}^{obs}$ with lowering pH symmetrically on both sides of the membrane in the presence of a leak current dissipating the membrane potential (Fang et al., 2007). A similar trend was subsequently shown with a mutant AdiC when the substrate on either side of membrane was Arg^+^ (Tsai et al., 2012). *F*_*max*_ of an obligatory 1:1 exchange is thermodynamically predetermined by the initial condition $\frac{n^{NR}\left(0\right)}{n^{R}\left(0\right)}$ or $\frac{r_{v}}{r_{c}}$ (Eq. 10) and should thus be independent of such a kinetics-influencing factor as pH. However, the rate to reach *F*_*max*_ depends on the kinetics and thus pH. Our model predicts an increase in the maximal peak fractional uptake with lowering pH (Fig. S11 B). Following the elimination of the apo path for conformational transitions, the model predicts the fractional uptake to approach the *F*_*max*_ predicted for a 1:1 exchanger, independent of pH (inset, Fig. S11 B).

### An integrative 4D model of AdiC

To create an integrated 4D transporter model for a chosen ligand condition, we used the temporal template shown in Fig. 11 A to connect the existing experiment-based structural models of individual conformational states. This 4D model is defined by: (1) the experiment-constrained 24-state kinetic model documented by the state diagram (Figs. 6 and S6), analytically expressed by the solutions to a system of 24 differential equations (Eq. S30) and fully defined by 60 *k*_i,j_ (Tables S1, S2, S3, and S4) plus 4 *K*_*D*i_ (Table S11); (2) the crystal structure models of the E_O_ and E_X_ states of an AdiC monomer (PDB 7O82 and 3L1L) and those of the I_O_ and I_X_ states of BasC and ApcT proper (PDB 6F2G and 3GIA) as AdiC’s proxies; and (3) the state-specific orientation information of helix-6A that directly links the corresponding structural and conformational states. To effectively exhibit the transporter model, we generated a video to illustrate the dynamic characteristics of conformational mechanism underlying the counter transport of Arg^+^ and Agm^2+^ (Video 2 for viewing in a small window). 13 frames of such a video are shown in Fig. 11 A. This integrative 4D model summarizes our collective understanding of the conformational dynamic mechanism underlying substrate transport in both spatial and temporal dimensions.

## Discussion

Our primary goals are to use AdiC as an example to demonstrate: (1) how to use the present microscopy method to determine a very large number of parameters that fully quantify a protein’s complex conformational behaviors in the temporal domain and (2) how to extract the underlying kinetic rate constants and equilibrium dissociation constants from these parameters, with which we can establish a model that quantitatively accounts for the observed conformational dynamics. These demonstrations have led to the determination of the 60 rate constants and 4 equilibrium dissociation constants that fully define a 24-state kinetic model of AdiC, whose analytic solution is derived in the supplemental text at the end of the PDF (Fig. 6; see also Fig. S12). The successful determination of such a complex conformation-kinetic mechanism of AdiC demonstrates the unprecedented resolving power of the present method.

The conformation-kinetic model is built from the polarization data, not optimized for its ability to describe transport-functional characteristics. However, given that a protein’s structural conformations are the physical determinants of its function, an adequate knowledge of its conformational kinetics should predict its function. Indeed, our conformation-kinetic model satisfactorily predicts relevant previous and present functional data of AdiC (Figs. 11 C, 12, 13, 14, 15, 16, S8, S9, S10, and S11).

Mechanistically, the present study reveals AdiC’s complex kinetics of transitioning among eight inherent states differing in conformation or free energy. These transitions occur spontaneously, depending on thermal energy. They enable AdiC to facilitate the transmembrane movement of Arg^+^ or Agm^2+^ and thus exhibit the substrate-concentration dependence. The concentration gradient of either Arg^+^ or Agm^2+^ or both would perturb the inherent probabilities of individual states (Eq. S32), altering the net transmembrane flux of substrate (Fig. 15).

The unexpected observation of AdiC transitioning among all resolved conformations bound with or without substrate is inconsistent with AdiC being an exchanger. To corroborate this finding, we performed radioactive flux assays and found: (1) AdiC-containing vesicles could hold a maximum uptake of radioactive substrate over several hours, only when the starting substrates concentration on both sides of vesicle membrane were much higher than K_D_ values; (2) the fractional uptake of radioactive substrate dropped over a long observation period, if the starting concentration of substrate in the bathing solution was lowered to near *K*_m_ values; and (3) under low substrate conditions, the observed fractional uptakes at *t* = 4 h were markedly lower than the theoretical *F*_*max*_ values predicted for a 1:1 exchanger but are generally predicted by our model (Fig. 15). As outlined in Results, our experimental findings and uniporter model are consistent with the relevant previous studies, all of which are incompatible with AdiC being a 1:1 exchanger. Nonetheless, with sufficiently high concentrations of substrates on both sides of the vesicle membrane, the utilization of the apo pathway for conformational changes in AdiC should be minimized, a condition that would cause AdiC to act like an exchanger (Fig. 14 and Fig. 15 A).

Biologically, enterobacteria acquire environmental Arg^+^ via several transporters (Charlier and Bervoets, 2019). In bacteria, Arg^+^ is decarboxylated, yielding Agm^2+^ and CO_2_. Eliminating CO_2_ lowers cytosolic acidity. If AdiC is a uniporter, it will facilitate the extrusion of excess Agm^2+^ converted from Arg^+^ acquired via multiple types of transporters, even when the environmental Arg^+^ concentration is low. Extrusion of divalent Agm^2+^ against the strongly negative membrane potential of bacteria is even harder than an exchange for a monovalent Arg^+^. To solve this problem, the bacteria resort to the electrical-shunt CLC channels (Iyer et al., 2002).

Motivated by the present study and a general technical need, we compared a monofunctional fluorophore with the standard bifunctional rhodamine and obtained comparable results with them. Attachment of a monofunctional fluorophore requires only a single cysteine, enabling the use of even a suitable native cysteine. The large collection of commercially available monofunctional fluorophores of various beneficial features may now be utilized, features including high quantum yield, long photobleaching time at a given excitation intensity, and different emission wavelengths. As such, multiple domains in a protein molecule can be tracked by labeling them with multiple fluorophores of different colors.

In summary, using the AdiC transporter, we have demonstrated the unprecedented capability of a high-resolution fluoroscence polarization microscopy system in acquiring the data required for solving a highly complex kinetic mechanism of transmembrane protein conformational dynamics. The acquisition of these data and their subsequent analysis have led to the determinations of 60 rate constants and 4 equilibrium dissociation constants. These determinations enabled us to establish an analytic conformation-kinetic model of 24 states expressed by a system of 24 differential equations that is fully constrained by the polarization data and can be used to qualitatively account for the conformational behaviors of AdiC for a given set of specific substrate conditions. This conformation-kinetic model, which has no adjustable parameters for a given substrate condition and pH, also satisfactorily predicts the previous and present observations of AdiC’s transport behaviors, even though the model building was uninfluenced by these transport behaviors. The transitions among all resolved conformations in the absence of substrates, which are detected from the polarization measurements, and the data yielded from previous relavent or present flux assays are all incompatible with the prevailing hypothesis that AdiC is a 1:1 exchanger. Here, the temporal information acquired in the polarization study is essential for our understanding the mechanisms of AdiC in 4D as well. Combining this temporal information and the existing structural information, we have illustrated how to construct an experiment-based integrative 4D model to capture and exhibit the spatiotemporal mechanisms of a facilitated transport of an amino acid and its metabolite. Thus, a combination of the present method and existing structural techniques serves as an effective means to help transition structural biology, including crystallography and cryo-EM, which has thus far been highly successful in the characterization of individual static structures, to an integrative form of dynamic structural biology. In addition, as a technical advance, we have successfully showcased the use of a monofunctional fluorophore in the present type of polarization microscopy study. Here, this monofunctional fluorophore and the standard bifunctional rhodamine yielded statistically comparable information, in terms of the number of resolvable conformational states and their relative orientations, probabilities, or kinetics. A use of monofunctional fluorophore will make the present method of an unprecedented kinetics-resolving capability more readily applied to the investigation of the conformational dynamics of many other types of protein.

## Supplementary Material

Table S1 shows the analysis results of lifetimes and transition probabilities for state C1 of AdiC in the absence or presence of Arg^+^ or Agm^2+^.

Table S2 shows the analysis results of lifetimes and transition probabilities for state C2 of AdiC in the absence or presence of Arg^+^ or Agm^2+^.

Table S3 shows the analysis results of lifetimes and transition probabilities for state C3 of AdiC in the absence or presence of Arg^+^ or Agm^2+^.

Table S4 shows the analysis results of lifetimes and transition probabilities for state C4 of AdiC in the absence or presence of Arg^+^ or Agm^2+^.

Table S5 shows the probabilities of the four conformational states of AdiC determined with bifunctional or monofunctional probes.

Table S6 shows the analysis results of lifetimes and transition probabilities for state C1 of monofunctional ATTO-550 labeled AdiC in the absence or presence of Arg^+^.

Table S7 shows the analysis results of lifetimes and transition probabilities for state C2 of monofunctional ATTO-550 labeled AdiC in the absence or presence of Arg^+^.

Table S8 shows the analysis results of lifetimes and transition probabilities for state C3 of monofunctional ATTO-550 labeled AdiC in the absence or presence of Arg^+^.

Table S9 shows the analysis results of lifetimes and transition probabilities for state C4 of monofunctional ATTO-550 labeled AdiC in the absence or presence of Arg^+^.

Table S10 shows the probabilities of states in the absence or presence Arg^+^ or Agm^2+^.

Table S11 shows the equilibrium dissociation constants of Arg^+^- or Agm^2+^-bound states.

Table S12 shows the equilibrium constants in the absence or presence of Arg^+^ or Agm^2+^.

Table S13 shows the observed and calculated k_max_ and K_m_ of Arg^+^ and Agm^2+^.

## Data Availability

The data used in the present study are available upon reasonable request.
